# Supramolecular cages as differential sensors for dicarboxylate anions: guest length sensing using principal component analysis of ESI-MS and ^1^H-NMR raw data†
†Electronic supplementary information (ESI) available: Experimental details of the principal component analysis, and ^1^H NMR and ESI-MS spectra. See DOI: 10.1039/c8sc05527k


**DOI:** 10.1039/c8sc05527k

**Published:** 2019-02-06

**Authors:** Carlo Bravin, Andrea Guidetti, Giulia Licini, Cristiano Zonta

**Affiliations:** a Department of Chemical Sciences , University of Padova , Via Marzolo 1 , 35131 Padova , Italy . Email: cristiano.zonta@unipd.it

## Abstract

Dynamic covalent libraries (DCLs) have been widely used in the development of differential sensors. Inspired by recent studies which use supramolecular recognition systems for sensing, we report the use of a tris(-pyridylmethyl)amine (TPMA)-based supramolecular cage as a differential sensor for dicarboxylate anions. In particular, a library of molecular cages constituted by linkers differing in size and flexibility was allowed to equilibrate toward a series of guests differing in molecular size. The differential system was able to discriminate a series of dicarboxylates depending on their chain length. This differentiation was evaluated through the application of the Principal Component Analysis (PCA) method using interpolated and raw data obtained from ESI-MS. Interestingly, while the ^1^H NMR spectra of the differential system did not allow for the discrimination of the analytes by the naked eye, PCA of the raw data from the spectra revealed information on the chain length of the guest and also on the odd-even character of the carbon chain.

## Abstract 2

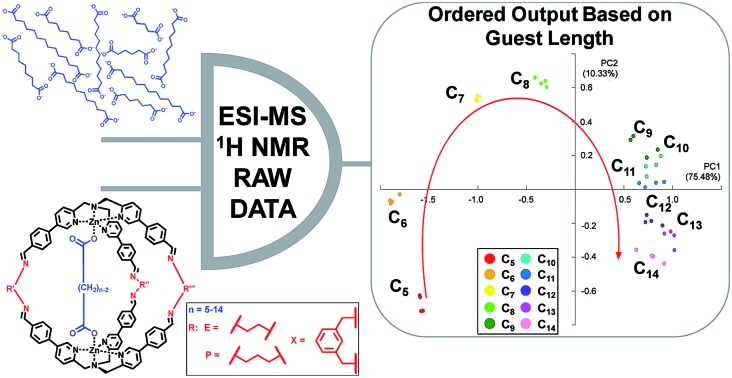
A differential sensor based on cages discriminate guests according to their length.

## Introduction

Differential sensing has recently become an analytical method capable of replacing traditional techniques based on direct molecular recognition.^1^–^9^ This approach takes inspiration from the olfactory sense of mammals which can discriminate between complex odorant mixtures without the necessity for highly specialized peripheral receptors.^10^,^11^ In broad terms, differential sensing employs a collection of low-selectivity receptors which give a peculiar signal for the analyte present in a solution. However, the large number of low-selectivity sensors is the key to overcome the problem of complicated matrixes.^12^–^18^ The discrimination made by the receptors is achieved by a characteristic “fingerprint” related to each system sensor analyte. This characteristic pattern is usually represented by an ensemble of parameters which are not easily described *via* simple calibration methods. In order to give an easy-to-read interpretation of the data collected, this sensing methodology was coupled to statistical analysis techniques, like discriminant analysis and principal component analysis (PCA), which are intensively used in many fields of academia and industry.^19^–^25^

**Fig. 1:**
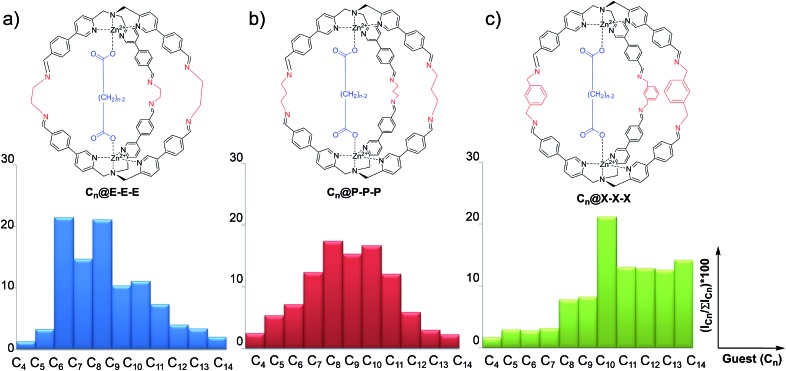
Selectivity profiles for cages (a) **C*_n_*@E-E-E**, (b) **C*_n_*@P-P-P** and (c) **C*_n_*@X-X-X** among the guest series ranging from C_4_ to C_14_ (*I*_C_*n*__/∑*I*_C_*n*__ represents the value of the relative intensity of the monoisotopic peak of each inclusion species among the guest series). The counteranion is perchlorate for the cage metals and triethylammonium for the carboxylate guests.

Among the possible chemical approaches, the capability of dynamic covalent libraries (DCLs) to respond to external signals has been widely used for the recognition and signalling of chemical stimuli.^26^–^28^ In particular, it is possible to employ complex systems characterized by multiple equilibria which can be perturbed by the presence of an analyte toward a particular product distribution. In this context, we have recently developed a new class of supramolecular structures,^29^–^33^ in particular molecular cages, which have been synthesized using imine dynamic covalent chemistry (DCC). These cages are obtained by self-assembly of modified tris(-pyridylmethyl)amine (TPMA) complexes and different diamine linkers.^34^–^36^ Variation of diamine linkers has allowed us to create a library of cages which have shown different selectivities toward a series of dicarboxylates (Fig. 1). ESI-MS studies, combined with ^1^H NMR, revealed that binding energies correlated the dimension of the cage with the length of the dicarboxylate (*viz.* the diamine linker length defines the distance between the two metal centres and, as a direct consequence, the preferred length of the guest included). For example, ethylenediamine linkers **E** in cage **C*_n_*@E-E-E** direct the system toward the preferential inclusion of adipate C_6_ as the best guest (Fig. 1a). On the other hand, the longer diamine linkers *m*-xylylenediamine **X** in cage **C*_n_*@X-X-X** lead to preferential binding of sebacate C_10_ (Fig. 1c).^34^

The peculiar capability of these cages to differentiate dicarboxylates by their length, combined with the dynamic nature of their formation, made this molecular system an ideal candidate for the development of a differential sensing array for dicarboxylate guests. Moreover, inspired by the systems reported by Anslyn^1^,^3^,^14^ and Alfonso,^37^–^43^ we checked if the differential sensing technique worked with raw data extracted directly from ESI-MS or ^1^H NMR analysis. This approach led to a system capable of discriminating dicarboxylate guests in the full range between C_5_ and C_14_ and unexpected results from the use of ^1^H NMR. The obtained results pave the way to another peculiar functional property, among the many other properties that supramolecular cages have shown so far.^44^–^53^

## Results and discussion

### Development of a differential sensing array using ESI-MS data

**Fig. 2:**
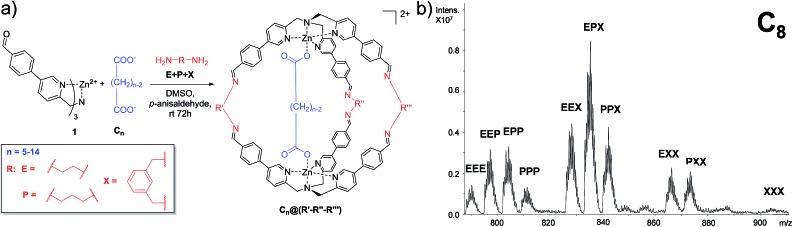
(a) DCL using different linkers **E**, **P** and **X**. The ratio between the complex, guest, linkers and *p*-anisaldehyde is 2 : 1 : 9 : 12 ([**1**] = 1.6 mM, [**C*_n_***] = 0.8 mM, and [**E**] = [**P**] = [**X**] = 2.4 mM [*p*-anisaldehyde] = 9.5 mM). (b) ESI-MS spectrum of the mixture in the presence of C_8_ as the guest after 72 hours.

In order to develop a differential sensing system, we set up a series of experiments in which the DCL is allowed to form different cages incorporating different linkers (*viz.* diamines) in the presence of a single guest (*viz.* dicarboxylate). In other words, rather than focusing on the binding selectivity of a series of guests towards a single cage, we investigated the formation of a cross-reactive array of multiple cages towards a single guest. In detail, the DCL consists of a solution containing one equivalent of dicarboxylate guests ranging from C_5_ to C_14,_ two equivalents of complex **1** and a mixture of the selected diamines **E**, **P**, and **X**. Under the experimental conditions, 3 equivalents of each diamine were added. In addition, to compensate the amine excess, *p*-anisaldehyde was added to the reaction mixture (Fig. 2a).

In a typical experiment, the dynamic system explores all the possible combinations of binding between the dicarboxylate under study and all possible molecular cages, and the system equilibrates thermodynamically toward the more stable inclusion cages distribution.

72 hours after mixing, the reaction mixture, diluted to an appropriate concentration for the MS technique, was injected into the ESI ion source. The typical MS trace displayed a series of *m*/*z* peaks corresponding to the different inclusion cages. For example, in Fig. 2b is reported the spectrum for the DCL experiment performed with C_8_ as the guest in which the di-charged peaks related to all ten possible formed cages are present.

**Fig. 3:**
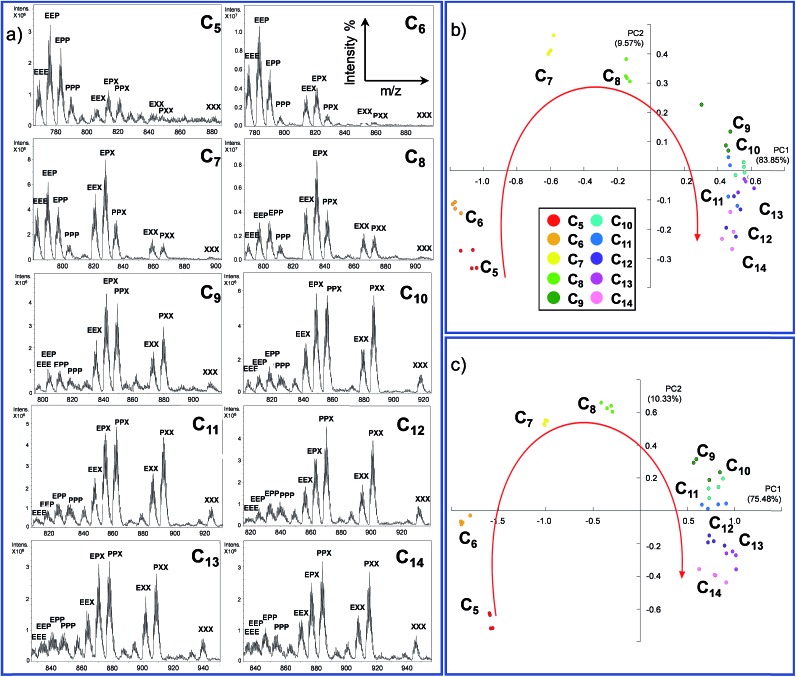
(a) ESI-MS of the DCL ranging from C_5_ to C_14_. Ten isotope clusters corresponding to the ten possible cages are present (doubly charged peaks for each included system). (b) Principal component analysis scores of the spectral data for the **E** + **P** + **X** experiment taking into account the normalized intensities of the monoisotopic peaks in four different experiments and (c) PCA of the normalized raw data of the MS spectra.

This experiment was extended to the series of guests ranging from C_5_ to C_14_, and the cage distribution is different for every single guest, as shown in Fig. 3a where the ESI-MS spectrum of each DCL experiment is reported (Fig. S1†).

At first glance, it can be reckoned that moving from C_5_ towards longer guests results in an increase of the presence of cages containing the longest diamine in the series *m*-xylylenediamine **X**. A flattening toward a similar distribution can also be noted in the case of longer guests (C_11_–C_14_). In other words, the DCL system responds similarly to longer dicarboxylate.

In order to gather more information, we employed principal component analysis (PCA), an unsupervised technique used to reduce the dimensionality of data space. As the data source, we extrapolated the relative monoisotopic peak of the included cages for each dicarboxylate guest (Table S1 and Fig. S2 in the ESI†). This chemometric tool allowed us to generate a new set of variables, named principal components (PCs), to explain the variance of the system. Each DCL experiment was repeated four times for each guest to evaluate the strength of the analytical method (see Section S3.1 in the ESI†).

In the PCA of our DCL system, the different dicarboxylates showed effective separation, allowing for discrimination based on the chain length (Fig. 3b). The two main principal components PC1 and PC2, which account for almost 95% of the whole variance, perform an arch disposition which is in accordance with the length of the guests. Although PC2 accounts for 9.56% of the variance and it is not easily associated into a chemical property of the system, the disposition of the data in the score plot could be interpreted by taking into account the “horseshoe effect” which is typical for a unimodal distribution.^54^,^55^ These results are also explained by the loading plot (Fig. S6 in the ESI†) which describes how positive values of PC1 correspond to the formation of cages containing longer linker **X**. In contrast, negative values indicate that mainly cages containing linker **E** are formed. However, the system has a strong tendency to promote the formation of mixed cages with the three different linkers **C*_n_*@E-P-X**, due to the stoichiometric ratio of diamines in the DCL system.

The considerations made directly for the ESI-MS data reflect the results obtained with the PCA; while short chains are well distributed in the first part of the arch, a smaller distinction is observed for longer guests.

However, extrapolation of the monoisotopic peak intensities is already a reduction of the data information. For this reason, we decided to perform the PCA on the normalized raw ESI-MS data (Section S3.2, ESI†).^56^ In this case, we introduce 126 points for each guest in the software four times. This reduces the data handling of the operator which could give rise to more information and thus better separation of the different guests. As expected, the results obtained by performing the PCA using the extrapolated monoisotopic peaks intensities (Fig. 3b) and the raw ESI-MS data are in agreement (Fig. 3c) in terms of the distribution of the different guests. However, the PCA obtained from the raw data analysis (i) increases the separation between the guests and (ii) allows for better clustering of the repeated analysis. In other words, the additional information present in the raw data allows for a better reproducibility of the measurement and it leads to a higher sensitivity of the method.

### Differential sensing with ^1^H NMR

PCA over ESI-MS data confirmed the discrimination capabilities of the cage sensor array. In addition, the PCA performed using raw data provides more information of the system allowing a better discrimination of the guest length.

However, the sensing capabilities were already noticeable from a “first glance” inspection of the ESI-MS trace. For this reason, we decided to investigate the response of our reactive array over ^1^H NMR spectroscopy, a technique that in principle should display very similar spectra to the cages formed.

To explore this possibility, we tested our sensing system in DMSO-d_6_ using the same experimental conditions described previously.

**Fig. 4:**
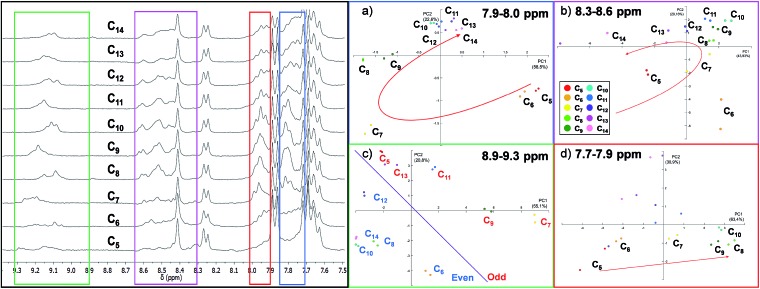
On the left: ^1^H NMR spectra (301 K, DMSO-d_6_) of the DCL in the presence of the guests ranging from C_5_ to C_14_. On the right: PCA performed considering four regions of the ^1^H NMR spectra (a) 7.9–8.0 ppm, (b) 8.3–8.6 ppm, (c) 8.9–9.3 ppm and (d) 7.7–7.9 ppm.

As expected, the resulting one dimensional ^1^H NMR spectra recorded after 72 hours related to the ten different experiments are similar and the differences are difficult to interpret since each reaction mixture contains at least ten different cages (Fig. 4 and S3 in the ESI†).

Using the raw data from ^1^H NMR, we built up a PCA taking into consideration the whole spectral region related to the cage signals (3.8–9.5 ppm). However, the whole spectra did not give any valuable information about the guest included (Fig. S7†). Therefore, we decided to focus our analysis on four different spectral windows which showed characteristic signal variations (Fig. 4).

The regions selected cover, respectively, the signals of the α-protons of the pyridine (8.9–9.3 ppm, green region in Fig. 4) and the imine protons of the cages (8.3–8.6 ppm, violet region) and two regions of the cage's aromatic protons related to the phenyl rings (7.9–8.0 ppm red region; 7.7–7.9 ppm blue region) (see ESI† Section S3.2).^57^

The PCA performed by analysing the data between 7.7 ppm and 7.9 ppm (Fig. 4d) shows that the system could discriminate between the length of different guests in the PC1/PC2 plane which accounts for 77% of the variance. In particular, only the intervals between C_5_ and C_10_ are well differentiated. A particular discrimination arises from the PCA performed between 8.9 and 9.3 ppm where a clear distinction between the odd and even length alkyl chains of the guests is observed by plotting PC1 *vs.* PC2 (Fig. 4c). The observed result is in agreement with previously characterized cages which report a lower chemical shift of the α-protons of the pyridine in the presence of even length guests.^35^ However, when the PCA was performed in the region between 7.9 ppm and 8.0 ppm, the ; length of the guests was discriminated and the PCA displays the above-mentioned “horseshoe effect” (Fig. 4a). A similar result was obtained considering the signals related to the imine protons of the cage (Fig. 4b).

To summarize, from the analysis of ^1^H NMR spectra which present indistinguishable differences, it is possible to gather information for the development of a sensor array. However, a careful evaluation of the spectral region should be taken. In particular, the large amount of numerical information corresponding to the whole spectra does not correspond to an increase in the discrimination capability of the sensor array, while this capability is achievable considering specific spectral regions.

### Projection of an unknown sample in the PCA space

In order to validate the recognition system, three unknown samples were chosen to cover the whole carboxylate lengths (C_5_, C_8_ and C_13_) and analyzed using MS and ^1^H NMR spectra. The corresponding experimental data were projected on the component space and compared to the original data using a prediction script (see Section S3.3 in the ESI†). Interestingly, the unknown samples are close in the vectorial space to previous validation samples. MS is able to predict the length of the system, and NMR confirms both its capability to predict the length and the odd-even character of the carboxylates (Fig. S12–S14 in the ESI†).

## Conclusions

In this work, a cross-reactive array of multiple cages for the differential sensing of guest length was developed. In order to achieve this objective, a series of experiments involving a mixture of three different linkers were performed and analyzed with ESI-MS and ^1^H NMR. The data obtained from the resulting spectra were used to form matrix data-sets which were statistically analyzed through PCA. The resulting scores for the ESI-MS spectra show that the system was able to discriminate guests according to their length. In particular, the array was able to efficiently distinguish all the guests in the full range from C_5_ to C_14_ using the monoisotopic peaks of the cages formed and the raw ESI-MS data as the input for the analysis. The PCA of ^1^H NMR spectra was able to distinguish odd and even guests, therefore providing information on structural features related to the guests. In addition, the prediction of unknown guests within the PCA space was evaluated and the results allow us to extend the use of the developed methodology also for the evaluation of unknown samples. More importantly, the developed methodology which extends the chemometric analysis to two techniques less studied in combination with PCA highlights the advantages and precautions in the case of the use of raw data.

## Conflicts of interest

There are no conflicts to declare.

## Supplementary Material

Supplementary information

## References

[cit1] Anslyn E. V. (2007). J. Org. Chem..

[cit2] Joyce L. A., Maynor M. S., Dragna J. M., da Cruz G. M., Lynch V. M., Canary J. W., Anslyn E. V. (2011). J. Am. Chem. Soc..

[cit3] Leung D., Kang S. O., Anslyn E. V. (2012). Chem. Soc. Rev..

[cit4] You L., Zha D., Anslyn E. V. (2015). Chem. Rev..

[cit5] Stewart S., Ivy M. A., Anslyn E. V. (2014). Chem. Soc. Rev..

[cit6] Adams M. M., Joyce L. A. and Anslyn E. V., Uses of Differential Sensing and Arrays in Chemical Analysis, in Supramolecular Chemistry, ed. P. A. Gale and J. W. Steed, 2012, 10.1002/9780470661345.smc021.

[cit7] Rakow N. A., Suslick K. S. (2000). Nature.

[cit8] Askim J. R., Mahmoudi M., Suslick K. S. (2013). Chem. Soc. Rev..

[cit9] Zardi P., Wurst K., Licini G., Zonta C. (2017). J. Am. Chem. Soc..

[cit10] Persaud K., Dodd G. (1982). Nature.

[cit11] Grosmaitre X., Santarelli L. C., Tan J., Luo M., Ma M. (2007). Nat. Neurosci..

[cit12] Zamora-Olivares D., Kaoud T. S., Dalby K. N., Anslyn E. V. (2013). J. Am. Chem. Soc..

[cit13] Zhang T., Anslyn E. V. (2006). Org. Lett..

[cit14] Jo H. H., Gao X., You L., Anslyn E. V., Krische M. J. (2015). Chem. Sci..

[cit15] Zhang T., Edwards N. Y., Bonizzoni M., Anslyn E. V. (2009). J. Am. Chem. Soc..

[cit16] Tobey S. L., Anslyn E. V. (2003). Org. Lett..

[cit17] Li X., Zamora-Olivares D., Diehl K. L., Tian W., Anslyn E. V. (2017). Supramol. Chem..

[cit18] Janzen M. C., Ponder J. B., Bailey D. P., Ingison C. K., Suslick K. S. (2006). Anal. Chem..

[cit19] Adams M. M., Anslyn E. V. (2009). J. Am. Chem. Soc..

[cit20] Brindle J. T., Antti H., Holmes E., Tranter G., Nicholson J. K., Bethell H. W. L., Clarke S., Schofield P. M., McKilligin E., Mosedale D. E., Grainger D. J. (2002). Nat. Med..

[cit21] Lever J., Krzywinski M., Altman N. (2017). Nat. Methods.

[cit22] Bro R., Smilde A. K. (2014). Anal. Methods.

[cit23] Zhang C., Suslick K. S. (2005). J. Am. Chem. Soc..

[cit24] Suslick B. A., Feng L., Suslick K. S. (2010). Anal. Chem..

[cit25] Lavigne J. J., Anslyn E. V. (2001). Angew. Chem., Int. Ed..

[cit26] You L., Long S. R., Lynch V. M., Anslyn E. V. (2011). Chem.–Eur. J..

[cit27] Rowan S. J., Cantrill S. J., Cousins G. R. L., Sanders J. K. M., Stoddart J. F. (2002). Angew. Chem., Int. Ed..

[cit28] Corbett P. T., Leclaire J., Vial L., West K. R., Wietor J.-L., Sanders J. K. M., Otto S. (2006). Chem. Rev..

[cit29] Scaramuzzo F. A., Licini G., Zonta C. (2013). Chem.–Eur. J..

[cit30] Scaramuzzo F. A., Badetti E., Licini G., Zonta C. (2017). Eur. J. Org. Chem..

[cit31] Badetti E., Dos Santos N. A. C., Scaramuzzo F. A., Bravin C., Wurst K., Licini G., Zonta C. (2018). RSC Adv..

[cit32] Badetti E., Wurst K., Licini G., Zonta C. (2016). Chem.–Eur. J..

[cit33] Berardozzi R., Badetti E., Carmo dos Santos N. A., Wurst K., Licini G., Pescitelli G., Zonta C., Di Bari L. (2016). Chem. Commun..

[cit34] Bravin C., Badetti C., Putreddy E., Pan R., Rissanen F., Licini K., Zonta G. (2018). Chem.–Eur. J..

[cit35] Bravin C., Badetti E., Scaramuzzo F. A., Licini G., Zonta C. (2017). J. Am. Chem. Soc..

[cit36] Bravin C., Licini G., Hunter C. A., Zonta C. (2019). Chem. Sci..

[cit37] Faggi E., Vicent C., Luis S. V., Alfonso I. (2015). Org. Biomol. Chem..

[cit38] Atcher J., Moure A., Bujons J., Alfonso I. (2015). Chem.–Eur. J..

[cit39] Valdivielso A. M., Puig-Castellví F., Atcher J., Solà J., Tauler R., Alfonso I. (2017). Chem.–Eur. J..

[cit40] Faggi E., Moure A., Bolte M., Vicent C., Luis S. V., Alfonso I. (2014). J. Org. Chem..

[cit41] Atcher J., Bujons J., Alfonso I. (2017). Chem. Commun..

[cit42] Corredor M., Carbajo D., Domingo C., Pérez Y., Bujons J., Messeguer A., Alfonso I. (2018). Angew. Chem., Int. Ed..

[cit43] Puig-Castellví F., Pérez Y., Piña B., Tauler R., Alfonso I. (2018). Anal. Chem..

[cit44] Rizzuto F. J., Kieffer M., Nitschke J. R. (2018). Chem. Sci..

[cit45] McConnell A. J., Wood C. S., Neelakandan P. P., Nitschke J. R. (2015). Chem. Rev..

[cit46] McConnell A. J., Haynes C. J. E., Grommet A. B., Aitchison C. M., Guilleme J., Mikutis S., Nitschke J. R. (2018). J. Am. Chem. Soc..

[cit47] Rizzuto F. J., Ramsay W. J., Nitschke J. R. (2018). J. Am. Chem. Soc..

[cit48] Cecot G., Marmier M., Geremia S., De Zorzi R., Vologzhanina A. V., Pattison P., Solari E., Fadaei Tirani F., Scopelliti R., Severin K. (2017). J. Am. Chem. Soc..

[cit49] Kostereli Z., Scopelliti R., Severin K. (2014). Chem. Sci..

[cit50] Cullen W., Misuraca M. C., Hunter C. A., Williams N. H., Ward M. D. (2016). Nat. Chem..

[cit51] Cullen W., Turega S., Hunter C. A., Ward M. D. (2015). Chem. Sci..

[cit52] Anderegg G., Wenk F. (1967). Helv. Chim. Acta.

[cit53] Jansze S. M., Cecot G., Severin K. (2018). Chem. Sci..

[cit54] Podani J., Miklós I. (2002). Ecology.

[cit55] Palmer M. W. (1993). Ecology.

[cit56] The raw ESI dataset contains 126 intensity *vs. m*/*z* corresponding to **C*_n_*@(R′-R′′-R′′′)**. In this set of data, the mass of the guest **C*_n_*** is removed and the data are subsequently normalized to the highest intensity

[cit57] Signal assignment was possible due to the previously characterized cages reported in ref. 34

