# Supplementary material for: Supramolecular cages as differential sensors for dicarboxylate anions: guest length sensing using principal component analysis of ESI-MS and 1H-NMR raw data
Source: Chem Sci. 2019 Feb 6;10(12):3523–8. doi: 10.1039/c8sc05527k (PMC6432344; doi:10.1039/c8sc05527k)
Supplement: Supplementary file 1 [file SC-010-C8SC05527K-s001.pdf]

Electronic Supplementary Material (ESI) for Chemical Science.
This journal is © The Royal Society of Chemistry 2019

Supporting Information for

Supramolecular Cages as Differential Sensors for Dicarboxylate Anions: Guest Length Sensing Using Principal Component Analysis of ESI-MS and 1H-NMR Raw Data

Authors: Carlo Bravin,[1] Andrea Guidetti,[1] Giulia Licini[1] and Cristiano Zonta[1]*

[1] Department of Chemical Sciences, University of Padova, via Marzolo 1, 35131 Padova

*Correspondence to: cristiano.zonta@unipd.it

Contents

Supramolecular Cages as Tool for Sensing of Guest Length Using Raw Data Analysis 1

1 General Methods 3

2 Synthesis and Characterization 4

3 Result and Discussions 5

3.1 General procedure for DCL formation with E-P-X linkers in the presence of the guests series 5

3.1.1 ESI-MS spectra 6

3.1.2 Selectivity profile of the C_n@(R-R’-R’’) cages 12

3.1.3 ^{1}H NMR spectra 13

3.2 General procedure for Data analysis with PCA 18

3.2.1 PCA of ESI-MS spectra 19

3.2.2 PCA of ^{1}H-NMR spectra 21

3.3 Prediction of an unknown by projection in the PCA space 24

3.3.1 PCA of ESI-MS spectra with additional data 25

3.3.2 PCA of ^{1}H-NMR spectra 26

1 General Methods

NMR spectra were recorded at 301 K on Bruker Avance-300. All the ^{1}H NMR spectra were referenced to residual isotopic impurity of DMSO-d_6 (2.50 ppm) or CD_3CN (1.98 ppm). The following abbreviations are used in reporting the multiplicity for NMR resonances; s=single, d=doublet, t= triplet, and m=multiplet. The NMR data were processed using MestReNova 10.0.2.
Low resolution electrospray ionization mass spectrometry LRMS (ESI-MS) experiments were carried out in positive mode with Agilent Technologies LC/MSD Trap SL AGILENT instrument (mobile phase Acetonitrile) with the following settings: Nebulizer = 20 psi, Dry gas = 5 L/min, Dry Temp = 325°C. The samples were infused from a syringe pump at the rate of 20 µL/min. MS peak intensity for each analysis was reported as monoisotopic mass and the data were processed with Data Explorer 4.2 and Openchrom 1.2.0 Community Edition. Principal Component Analysis were performed with MatLab R2017a.
Chemicals were purchased from Sigma-Aldrich, TCI, or Acros and used without further purification.

2 Synthesis and Characterization

Compounds **C_n@E-E-E, C_n@P-P-P, C_n@X-X-X** and complex **1** have been synthesized and fully characterized (^{1}H NMR, 2D NMR, MS analysis) accordingly to: C. Bravin, E. Badetti, F.A. Scaramuzzo, G. Licini, C. Zonta. *J. Am. Chem. Soc.* **2017**; *139*, 6456-6460 and C. Bravin, E. Badetti, R. Puttreddy, F. Pan, K. Rissanen G. Licini, C. Zonta. *Chem. Eur. J.* **2018**; *24*, 2936.

3 Result and Discussions

3.1 General procedure for DCL formation with E-P-X linkers in the presence of the guests series

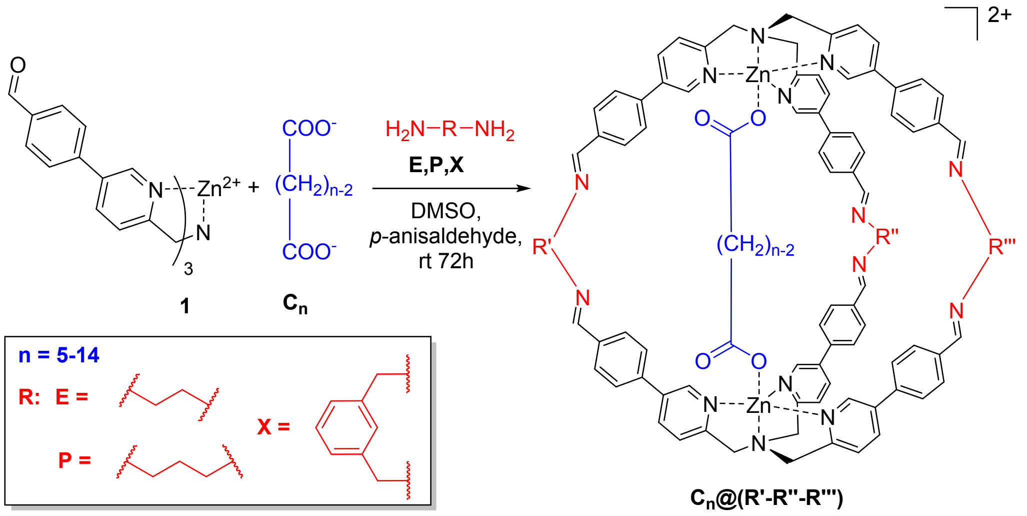

In a small flask were added 500 μl of a 0.002 M solution of complex **1** (0.001 mmol, 2 eq.), 25 μl of a 0.02 M solution of dicarboxylate $\mathbf{C_n}$ (0.0005 mmol, 1 eq.), 30 μl of a 0.2 M solution of *p*-anisaldehyde (0.006 mmol, 12 eq.) and 75 μl of a 0.02 M solution containing diamines **E**, **P** and **X** (0.0015 mmol, 3 eq. for each diamine) in DMSO. The mixture was left 3 days at room temperature and checked via ESI-MS (50 μl of the experiment mixture diluted in 3 ml of CH_3CN). The same experimental procedure was adopted for NMR experiment preparing the solution in DMSO-d_6.

3.1.1 ESI-MS spectra

The spectral window containing all the di-charged peaks related to the cage species **C_n@1(R-R'-R'')** for each guest of the **C_n** series. The symbols **E**, **P** and **X** represent the number of diamine linkers present in the structure (**E**=EDA, **P**=PDA **X**=mXDA) while **C_n** represents the guest used in each experiment. In Figure S1 are reported the ESI MS spectra of the DCL formation while in Figure S2 are reported the corresponding selectivity profile for each experiment considering the relative intensity of the *m/z* peaks (Table S1). In Figure S3 are reported the corresponding ^{1}H NMR spectra.

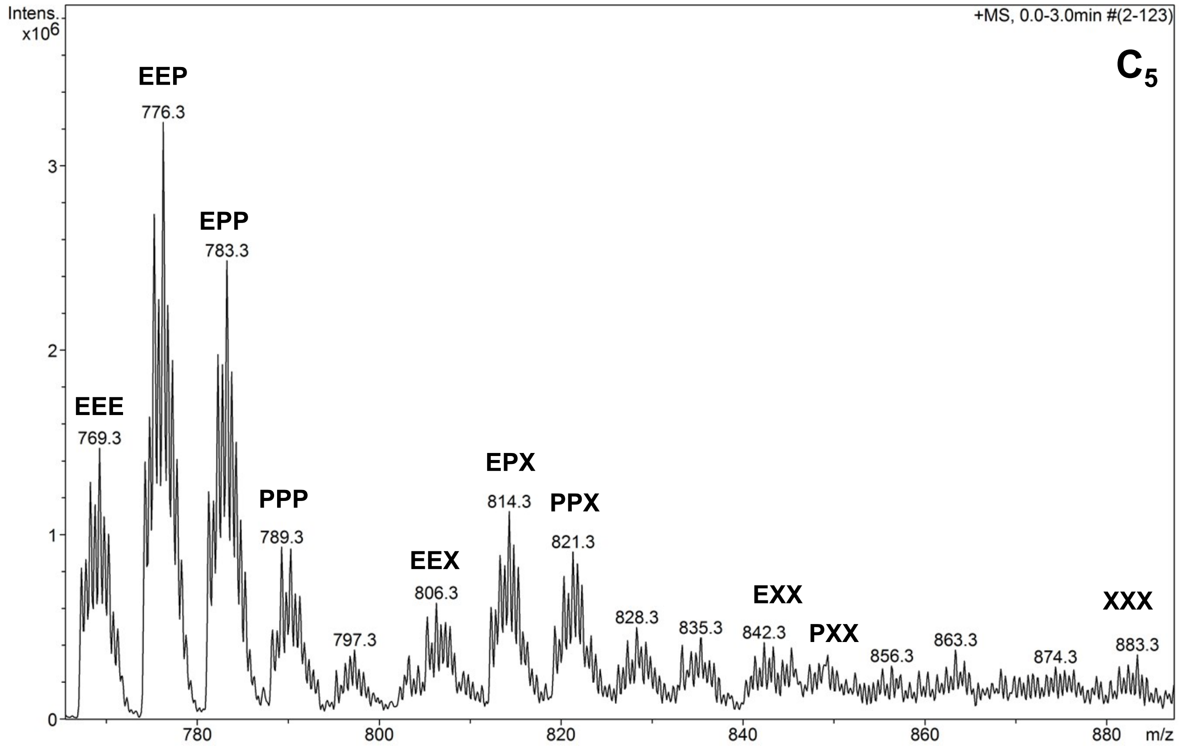

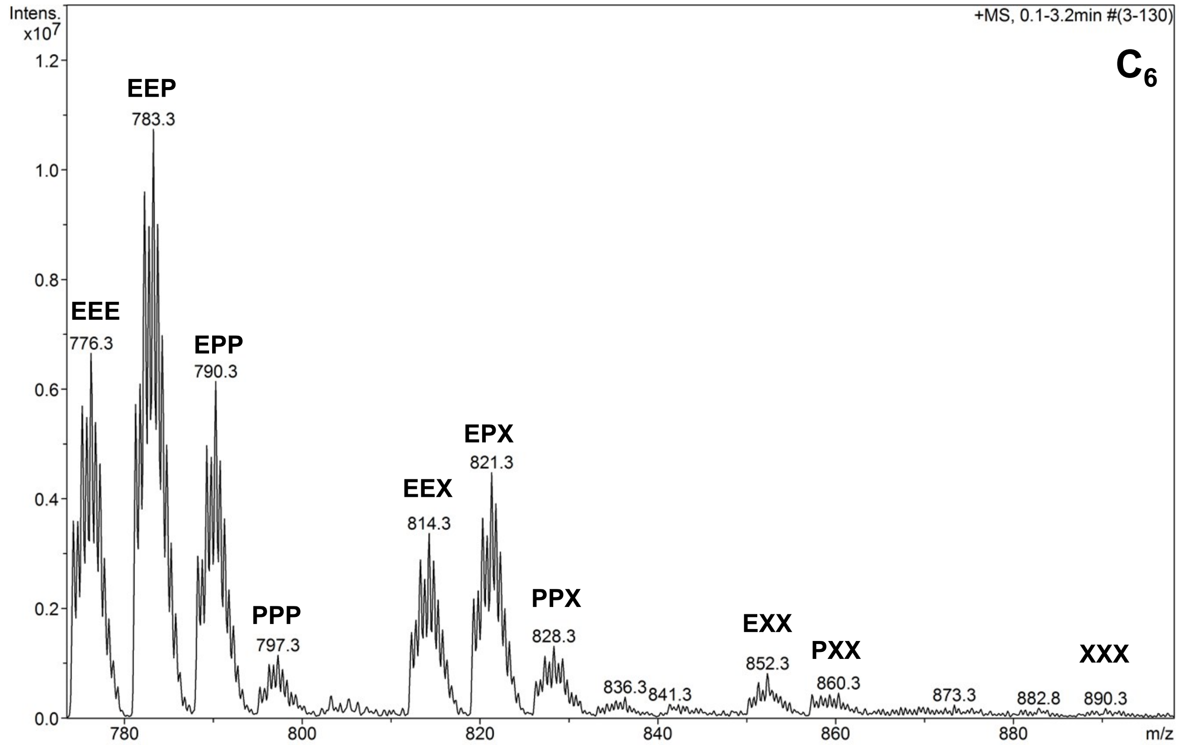

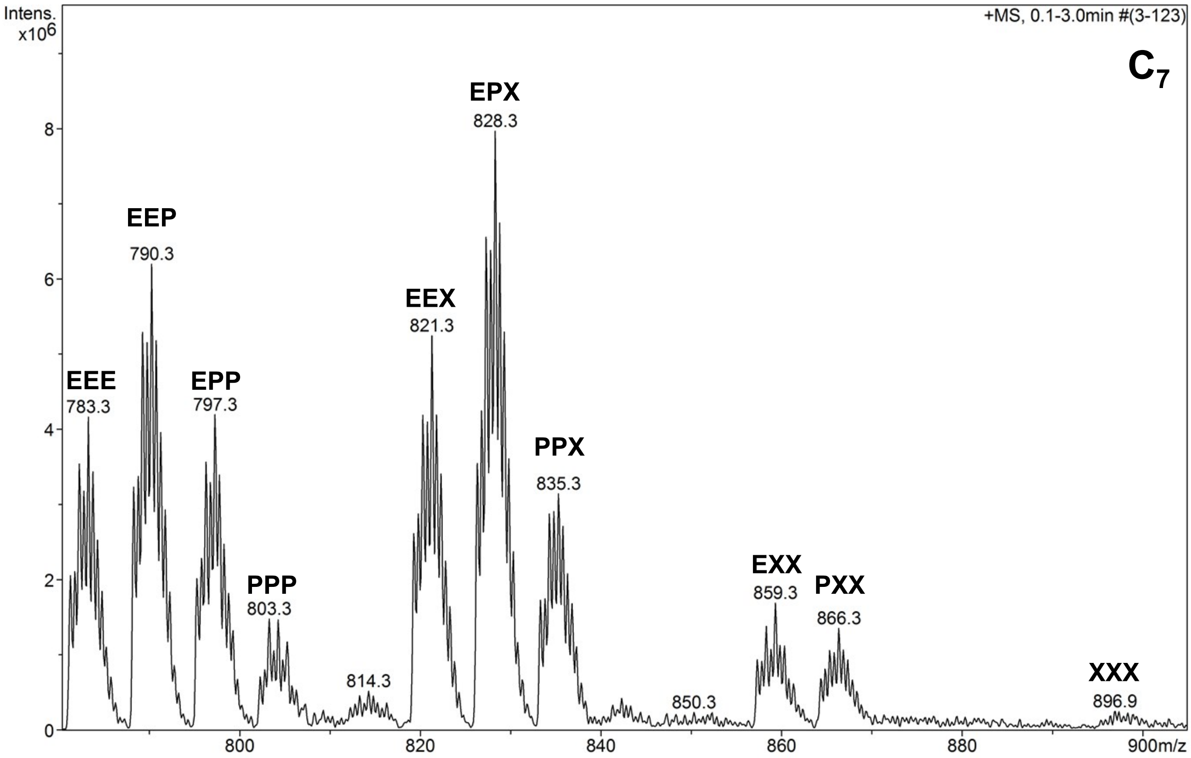

Intens. x10^6
+MS, 0.1-3.0min #(3-123)
C7
EEE 783.3
EEP 790.3
EPP 797.3
PPP 803.3
814.3
EEX 821.3
EPX 828.3
PPX 835.3
850.3
EXX 859.3
PXX 866.3
XXX 896.9
0
2
4
6
8
800
820
840
860
880
900m/z

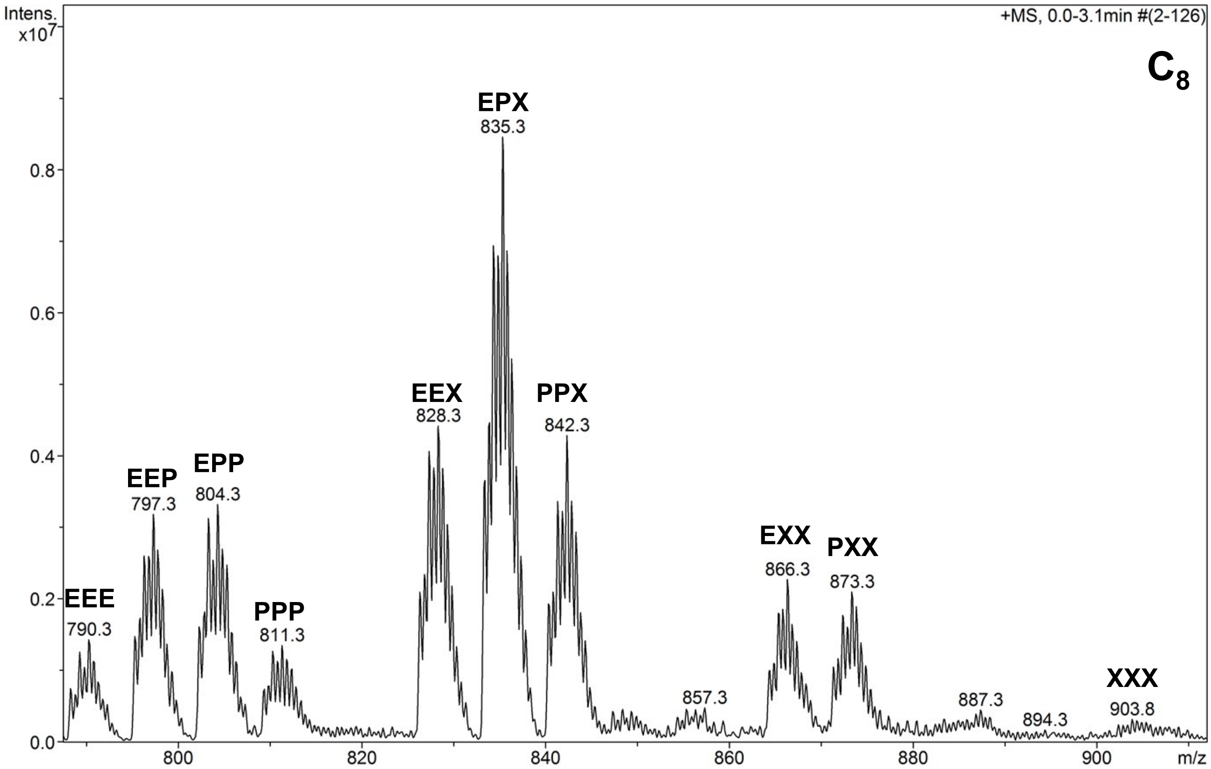

Intens. x10^7
+MS, 0.0-3.1min #(2-126)
C8
EEE 790.3
EEP 797.3
EPP 804.3
PPP 811.3
EEX 828.3
EPX 835.3
PPX 842.3
857.3
EXX 866.3
PXX 873.3
887.3
894.3
XXX 903.8
0.0
0.2
0.4
0.6
0.8
800
820
840
860
880
900
m/z

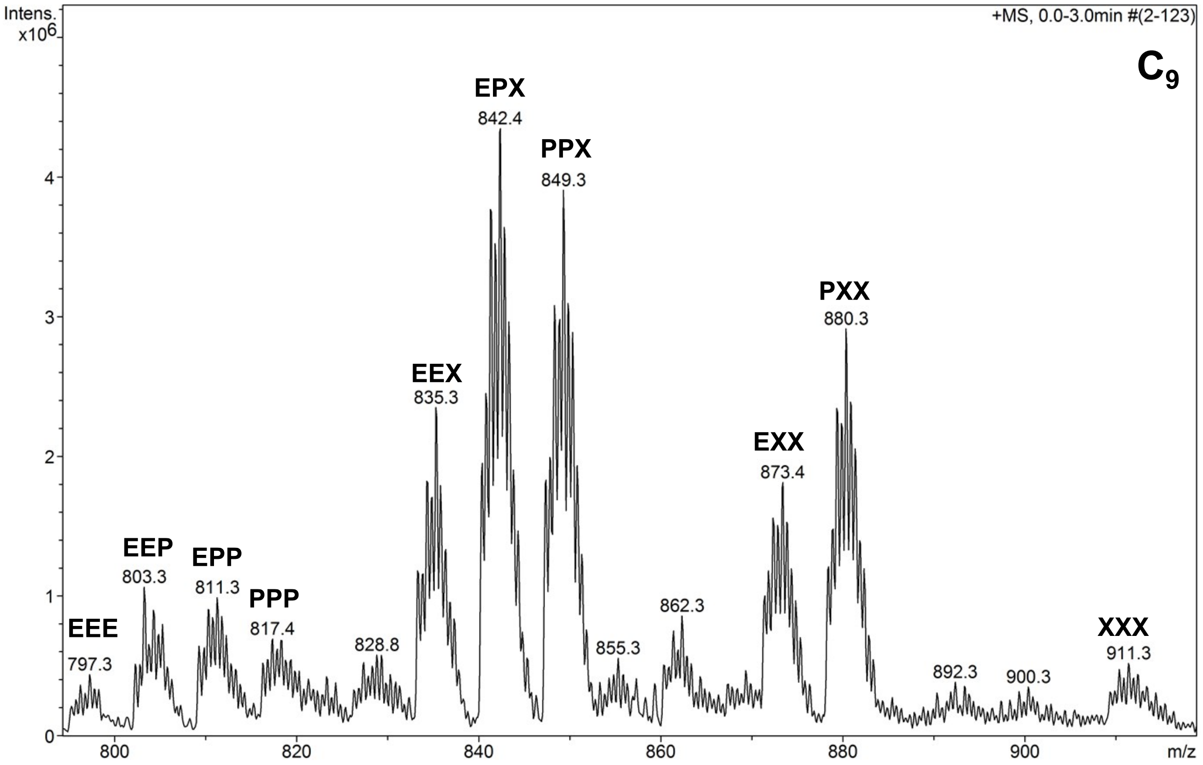
Intens.
x10^6
+MS, 0.0-3.0min #(2-123)
C9
EPX 842.4
PPX 849.3
PXX 880.3
EEX 835.3
EXX 873.4
EEP 803.3
EPP 811.3
PPP 817.4
EEE 797.3
828.8
855.3
862.3
892.3
900.3
XXX 911.3
m/z

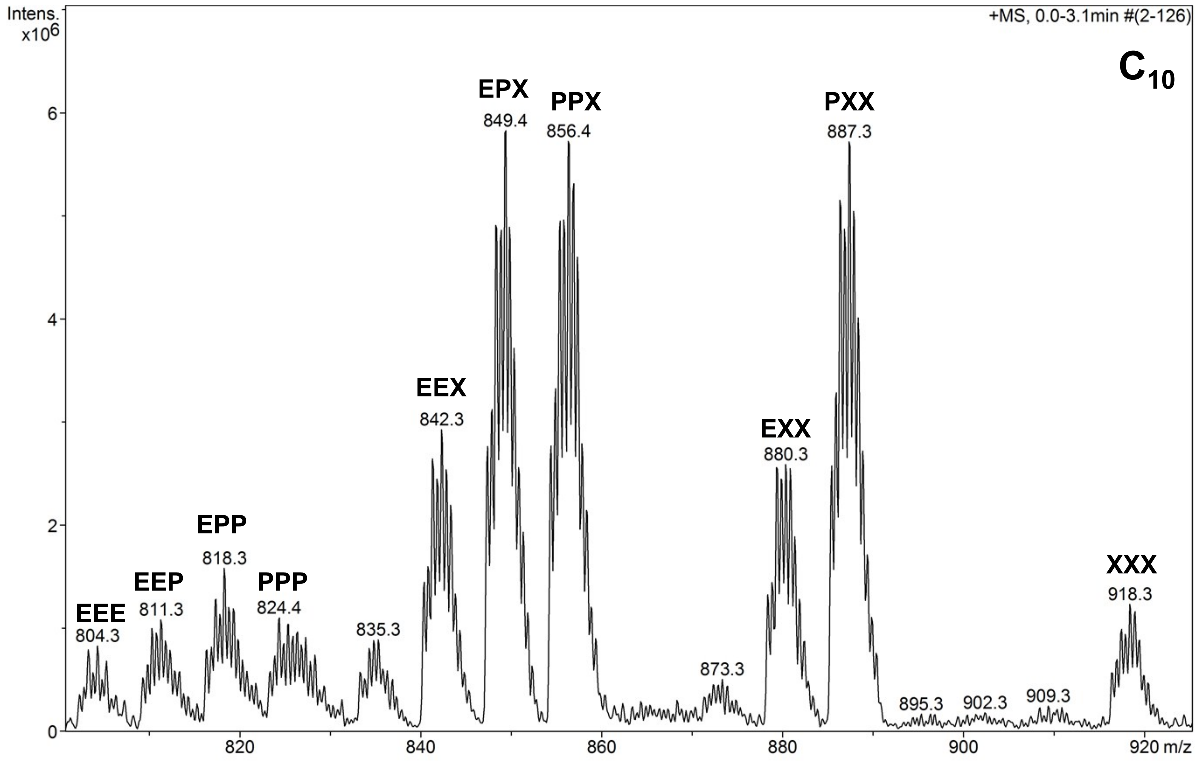
Intens.
x10^6
+MS, 0.0-3.1min #(2-126)
C10
EPX 849.4
PPX 856.4
PXX 887.3
EEX 842.3
EXX 880.3
EPP 818.3
EEP 811.3
PPP 824.4
EEE 804.3
835.3
873.3
895.3
902.3
909.3
XXX 918.3
m/z

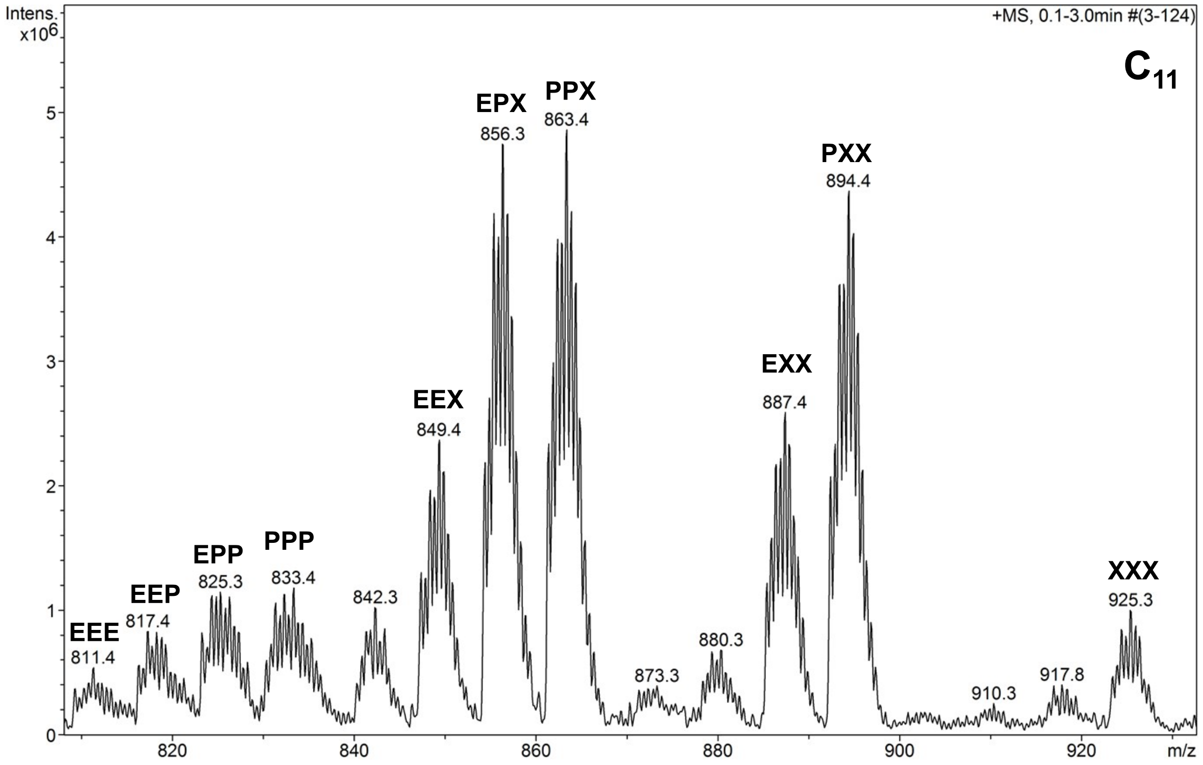
Intens.
x10^6
+MS, 0.1-3.0min #(3-124)
C$_{11}$
EEE 811.4
EEP 817.4
EPP 825.3
PPP 833.4
842.3
EEX 849.4
EPX 856.3
PPX 863.4
873.3
880.3
EXX 887.4
PXX 894.4
910.3
917.8
XXX 925.3
m/z

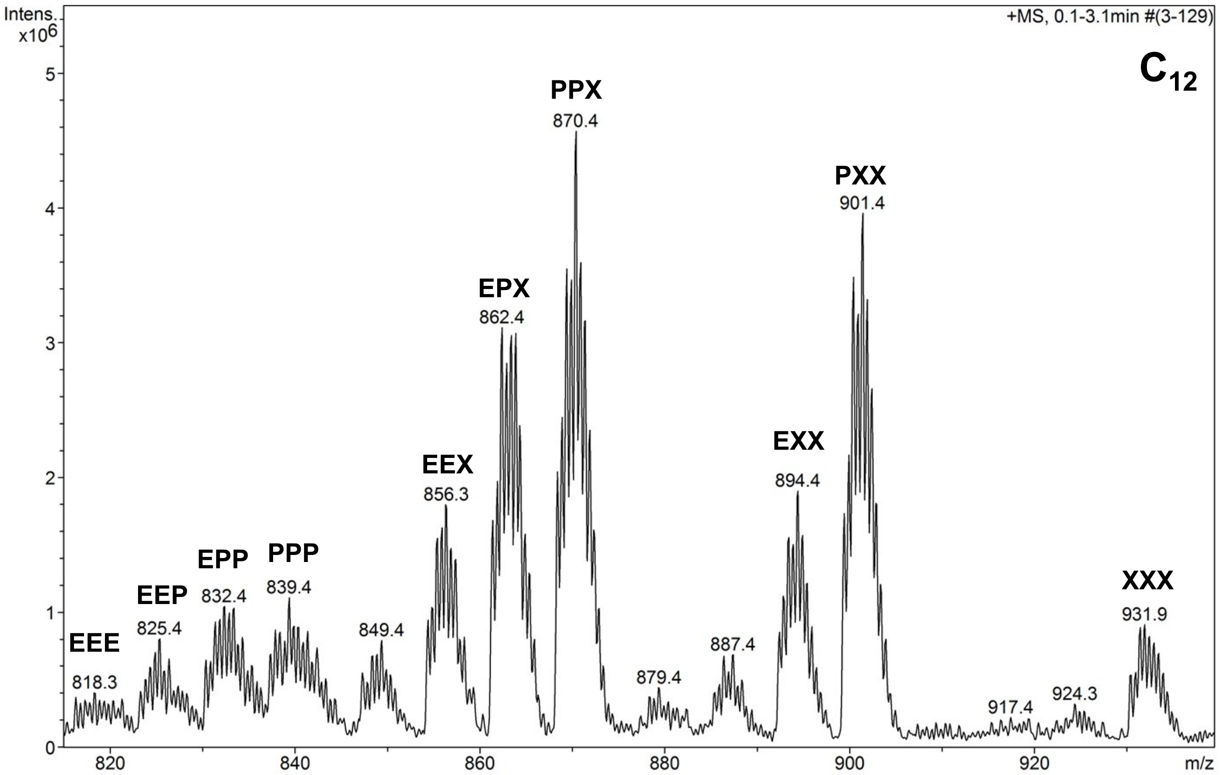
Intens.
x10^6
+MS, 0.1-3.1min #(3-129)
C$_{12}$
EEE 818.3
EEP 825.4
EPP 832.4
PPP 839.4
849.4
EEX 856.3
EPX 862.4
PPX 870.4
879.4
887.4
EXX 894.4
PXX 901.4
917.4
924.3
XXX 931.9
m/z

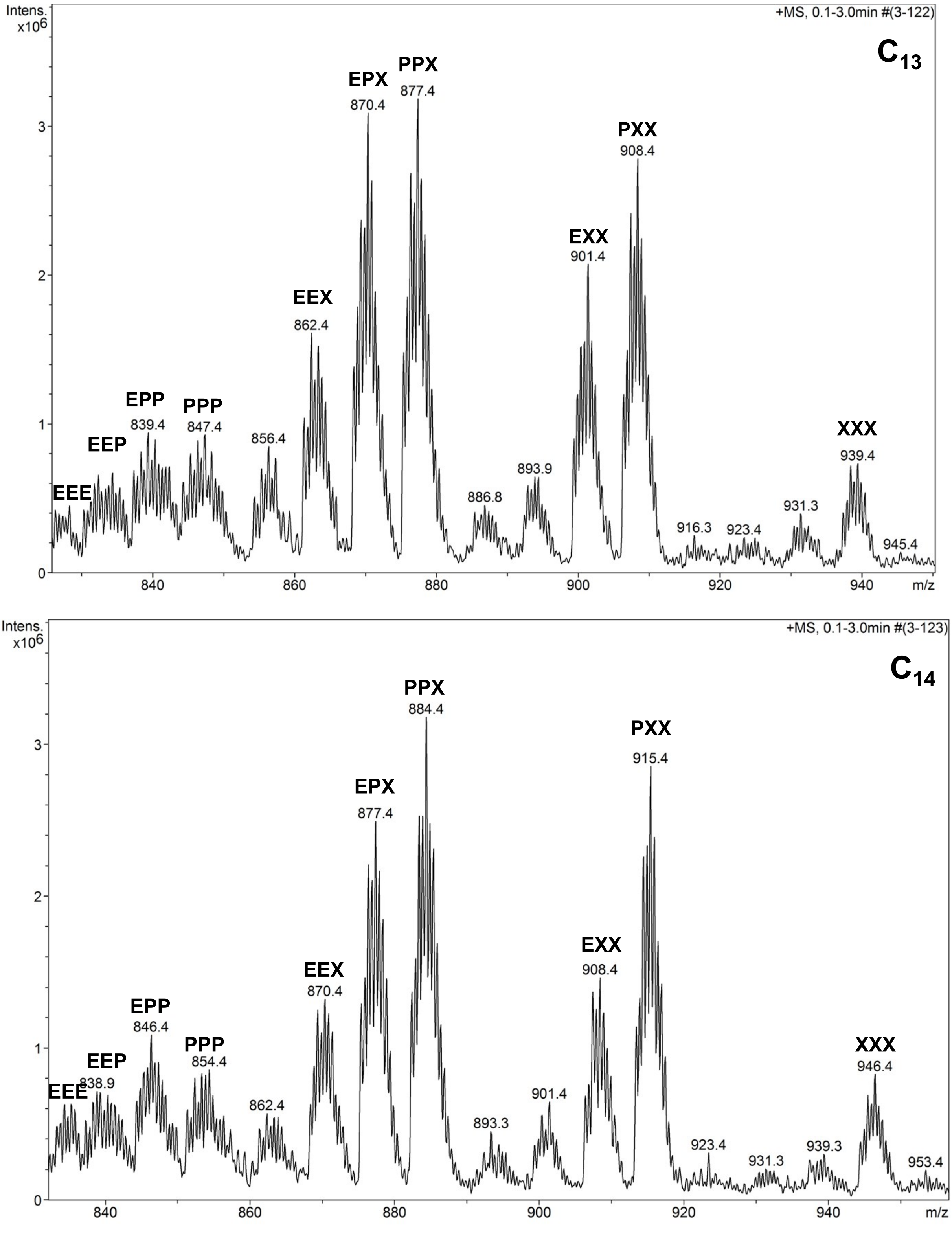

Figure S1 a) ESI-MS spectra of the **E+P+X** linkers experiments towards the guests series $\mathbf{C_5}$-$\mathbf{C_{14}}$.

The values of relative intensity $I_{Cn}/\sum I_{Cn}$ for each characteristic peak in the scrambling experiment and their standard deviation are reported in Table S1. The error reported for the ESI-MS experiment are in agreement with our previous work (see ref. [26] in the article). The measures were repeated four times. The values obtained are showed in the histograms showed in Figure S2.

	$I_{Cn}/\sum I_{Cn}$				
Cage / Guest	1(EEE)	1(EEP)	1(EPP)	1(PPP)	1(EEX)
C_5	0.131±0.006	0.264±0.006	0.210±0.010	0.080±0.007	0.053±0.005
C_6	0.187±0.008	0.311±0.011	0.170±0.008	0.035±0.002	0.091±0.010
C_7	0.110±0.004	0.174±0.007	0.118±0.008	0.039±0.003	0.147±0.012
C_8	0.046±0.004	0.098±0.008	0.108±0.014	0.052±0.002	0.143±0.001
C_9	0.025±0.003	0.047±0.002	0.058±0.006	0.045±0.005	0.119±0.005
C_{10}	0.027±0.001	0.041±0.002	0.052±0.005	0.039±0.003	0.104±0.003
C_{11}	0.027±0.002	0.040±0.003	0.054±0.004	0.051±0.006	0.094±0.004
C_{12}	0.025±0.002	0.043±0.005	0.061±0.004	0.053±0.004	0.095±0.003
C_{13}	0.025±0.002	0.042±0.003	0.061±0.006	0.052±0.005	0.099±0.003
C_{14}	0.025±0.002	0.052±0.003	0.077±0.002	0.060±0.002	0.084±0.010
Cage / Guest	1(EPX)	1(PPX)	1(EXX)	1(PXX)	1(XXX)
C_5	0.097±0.007	0.073±0.001	0.036±0.006	0.026±0.002	0.031±0.003
C_6	0.124±0.007	0.040±0.008	0.024±0.001	0.014±0.001	0.005±0.001
C_7	0.222±0.009	0.095±0.002	0.045±0.004	0.040±0.002	0.009±0.001
C_8	0.244±0.017	0.144±0.006	0.075±0.004	0.079±0.006	0.011±0.001
C_9	0.213±0.012	0.207±0.006	0.106±0.003	0.150±0.019	0.030±0.005
C_{10}	0.203±0.005	0.202±0.011	0.095±0.007	0.193±0.006	0.044±0.001
C_{11}	0.197±0.002	0.212±0.015	0.105±0.009	0.176±0.005	0.045±0.002
C_{12}	0.166±0.003	0.215±0.012	0.100±0.003	0.191±0.003	0.051±0.005
C_{13}	0.177±0.008	0.194±0.006	0.130±0.004	0.168±0.004	0.051±0.001
C_{14}	0.158±0.008	0.202±0.002	0.105±0.009	0.181±0.011	0.057±0.003

Table S1 Relative intensity values of inclusion complexes **C_n@(R-R'-R'')** and relative standard deviation, calculated as the average of three peaks of each cluster for all the competition experiments.

3.1.2 Selectivity profile of the C_n@(R-R'-R'') cages

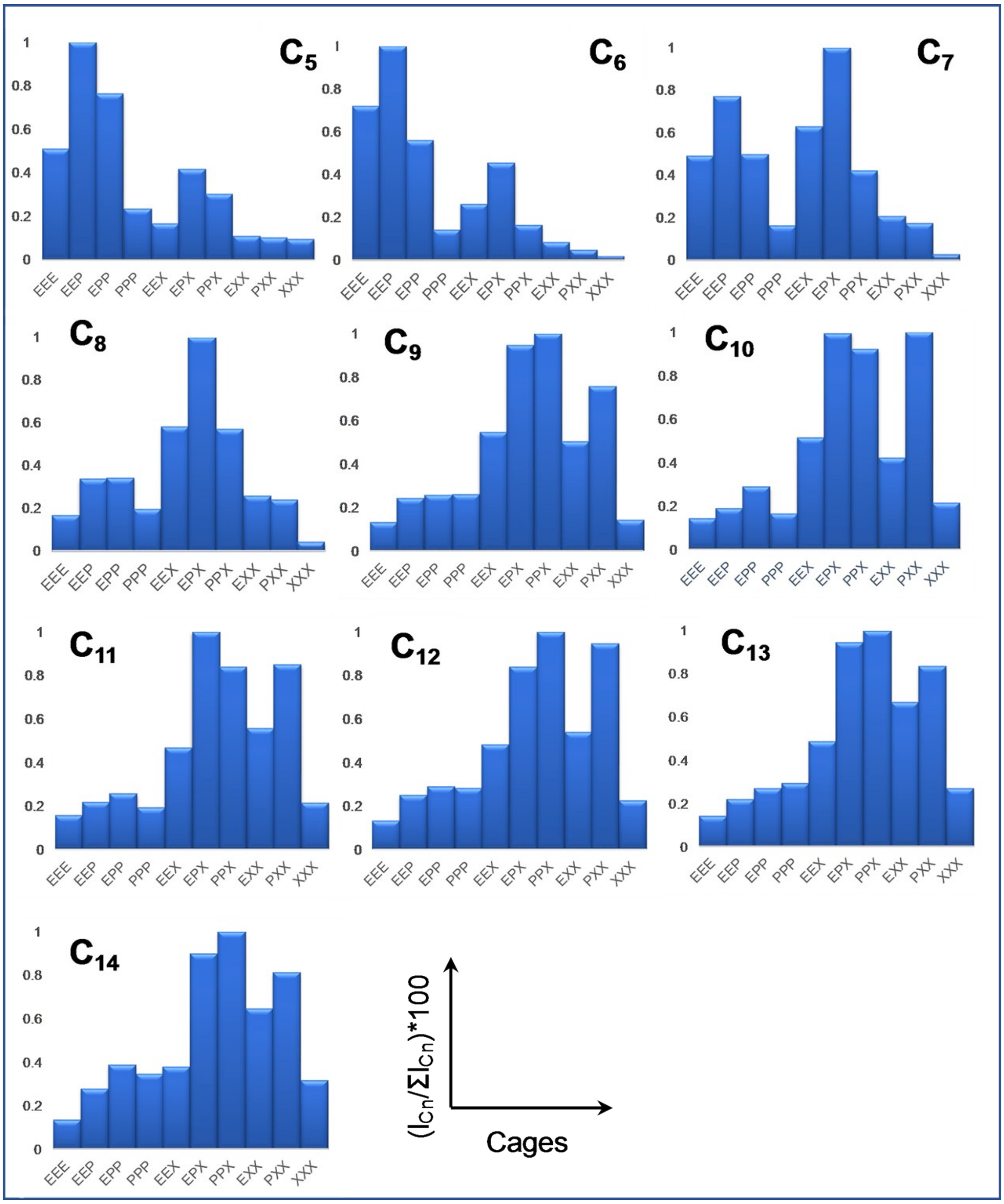

Figure S2 ESI-MS selectivity profiles for the **E+P+X** linkers experiments towards the guests series $\mathbf{C_5}$-$\mathbf{C_{14}}$.

3.1.3 ^{1}H NMR spectra

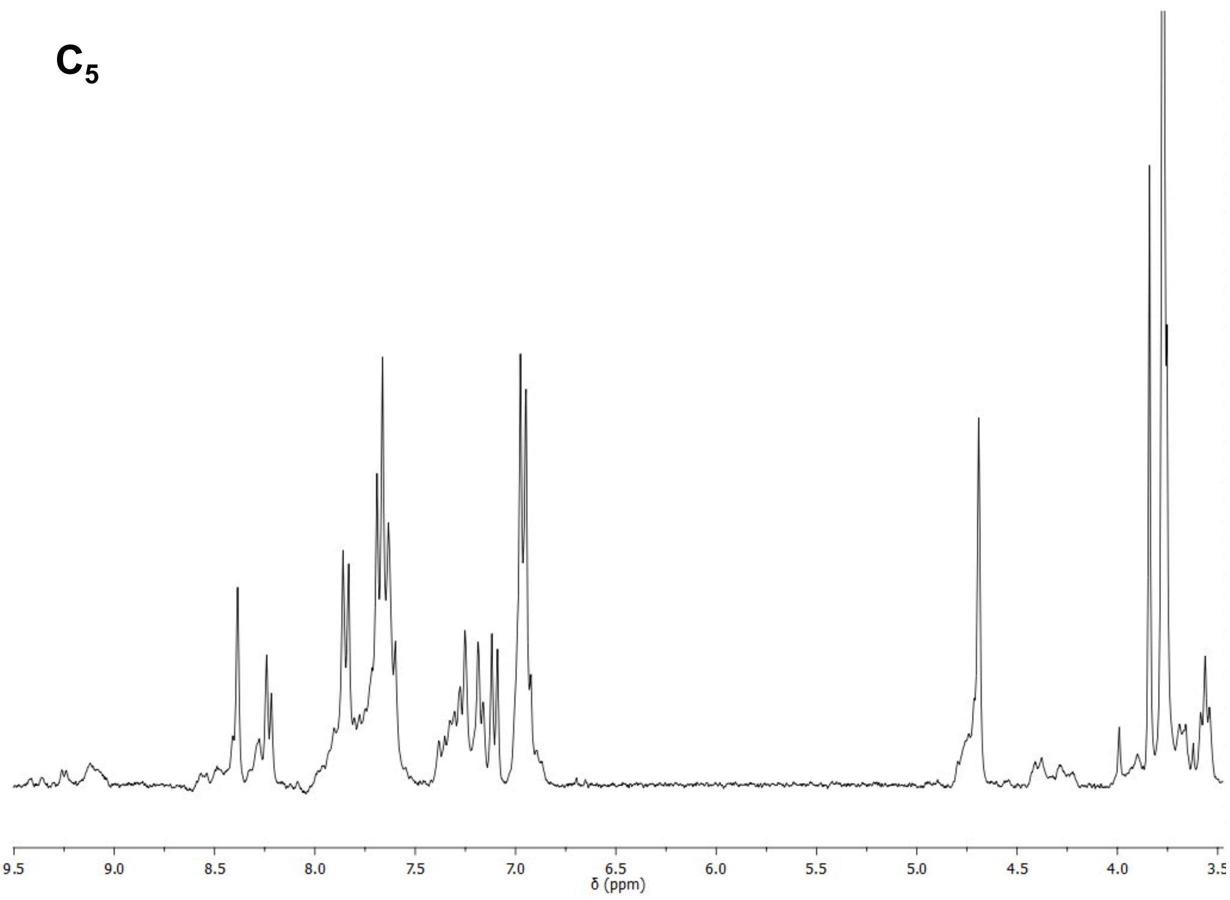

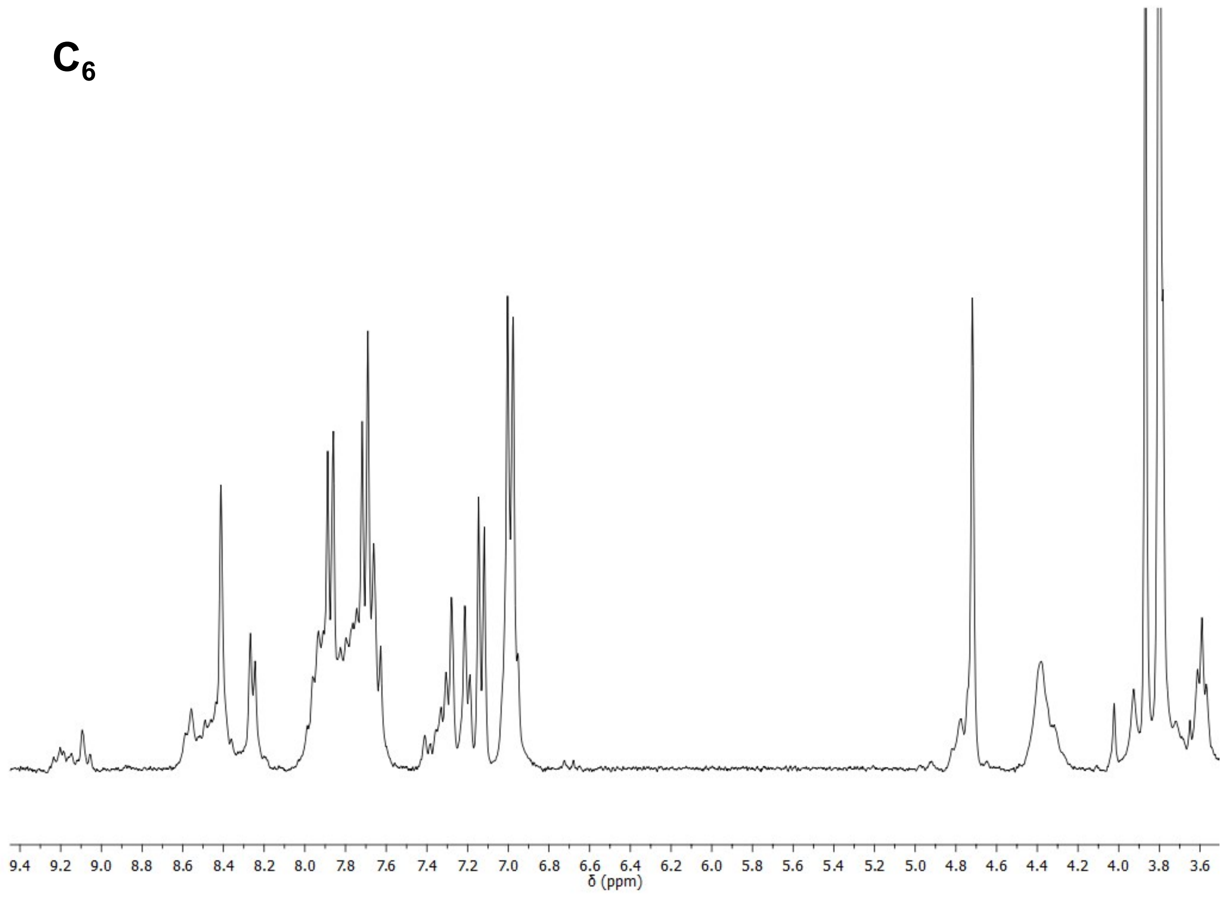

C_7

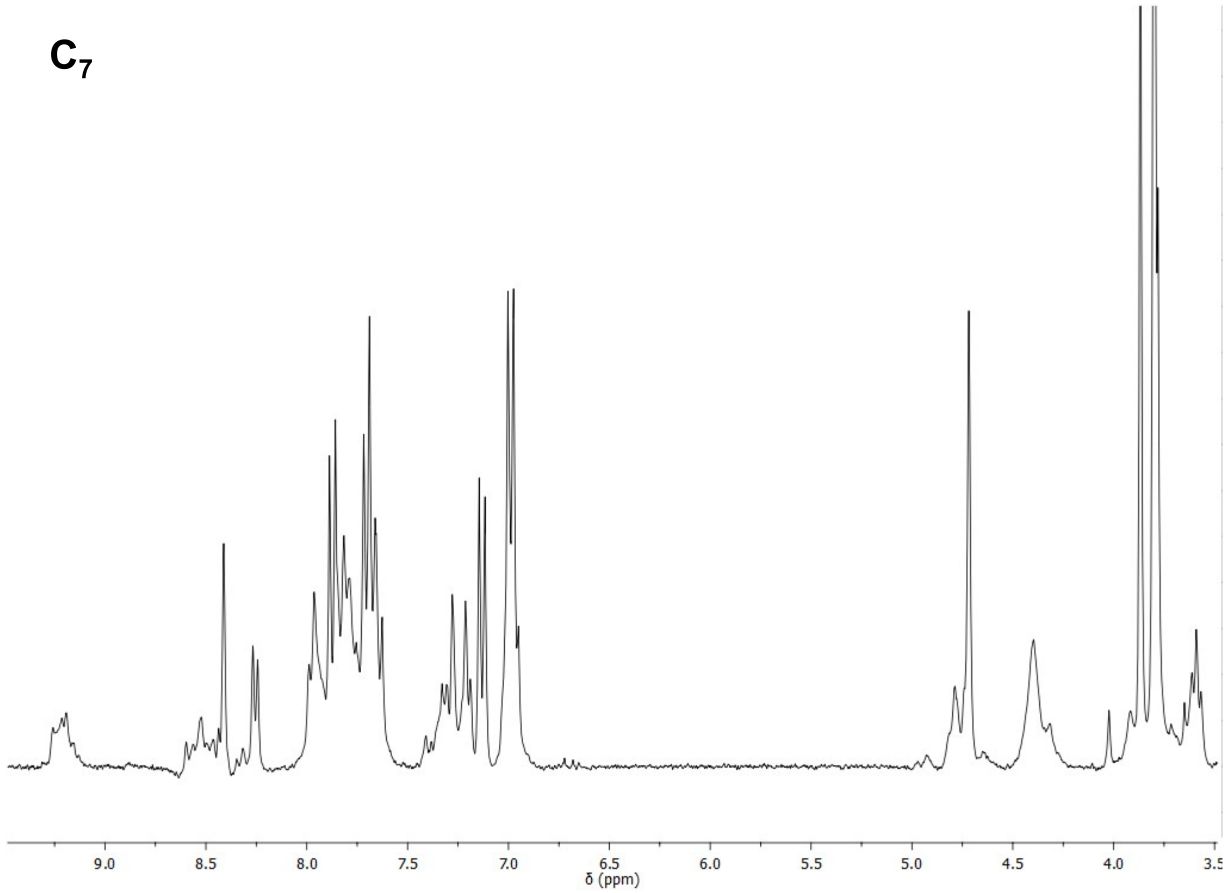
9.0
8.5
8.0
7.5
7.0
6.5
6.0
5.5
5.0
4.5
4.0
δ (ppm)

C_8

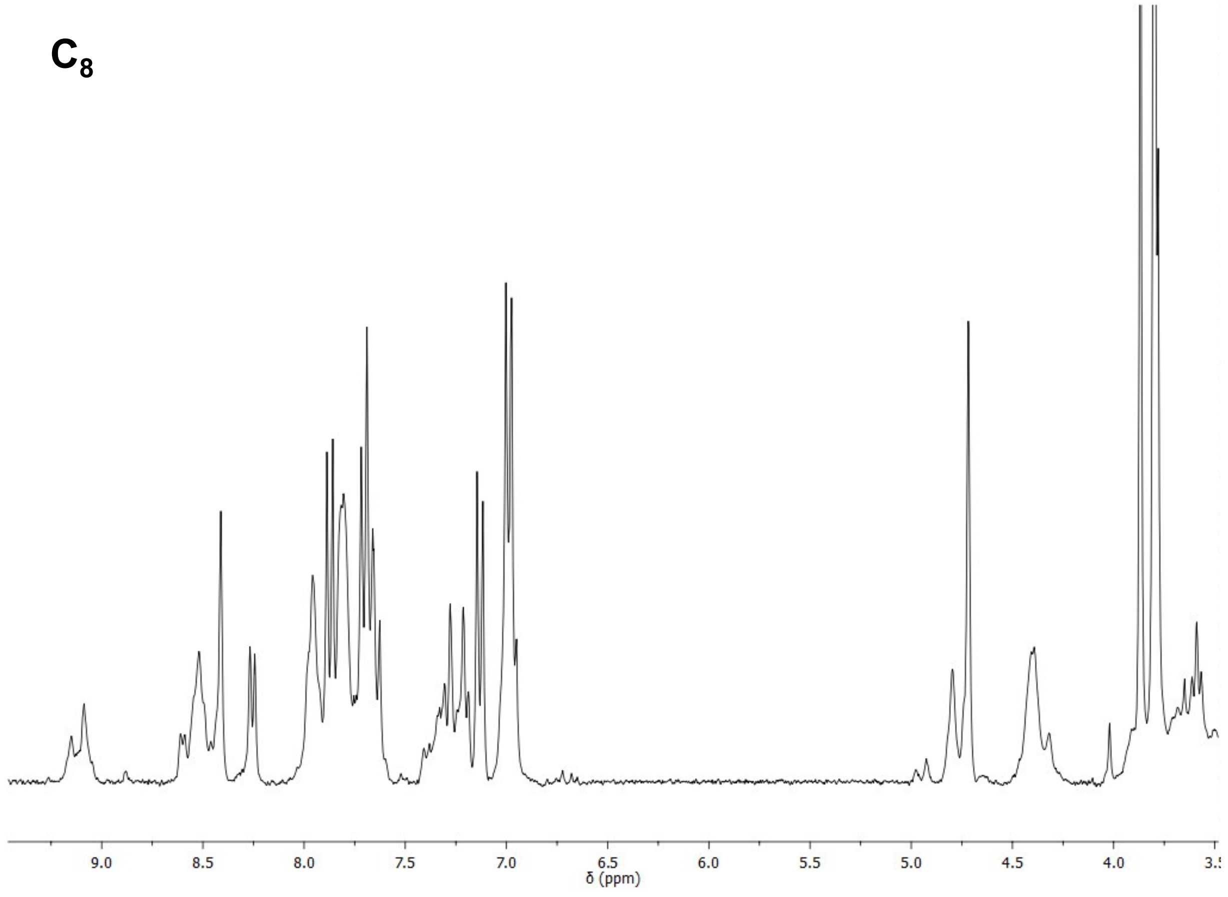
9.0
8.5
8.0
7.5
7.0
6.5
6.0
5.5
5.0
4.5
4.0
δ (ppm)

C_9

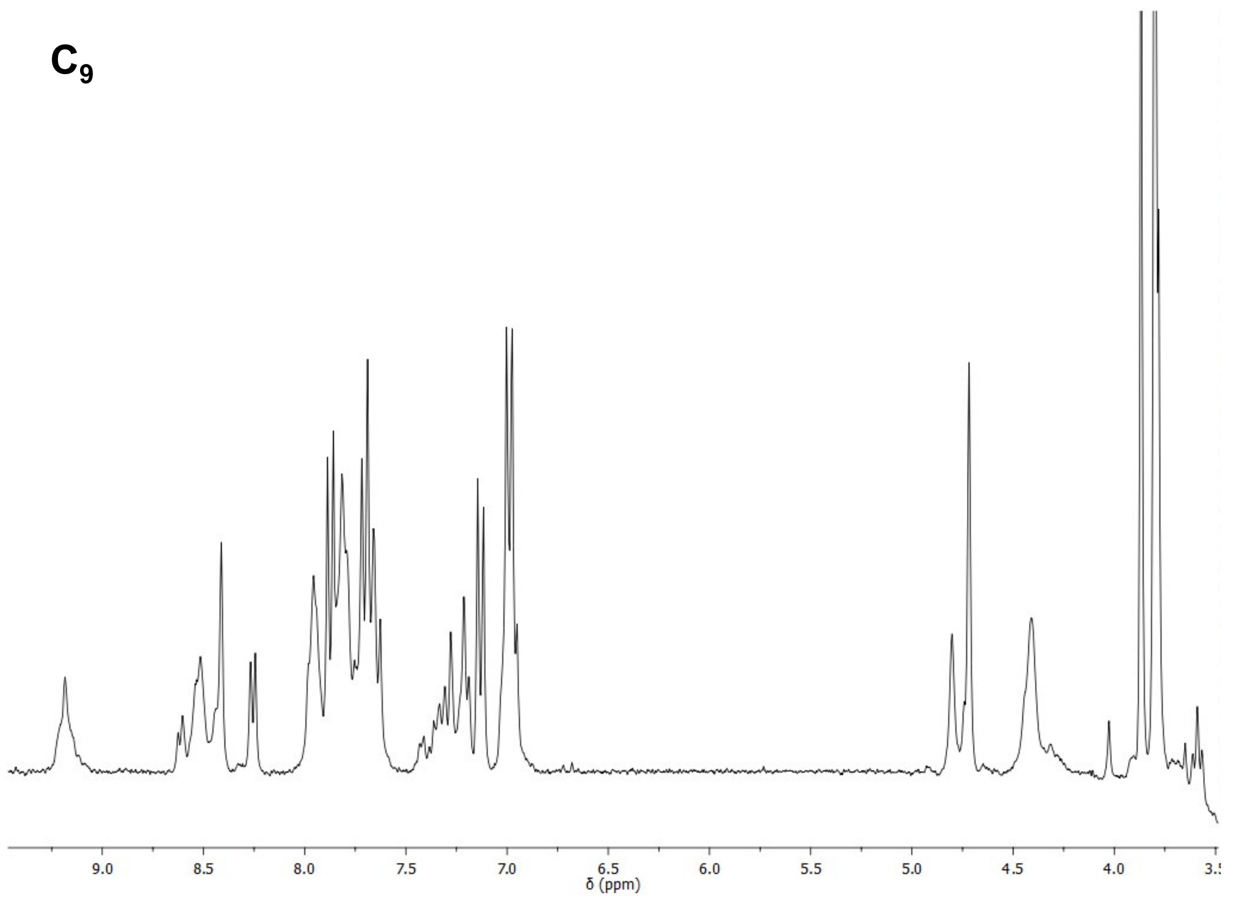
9.0
8.5
8.0
7.5
7.0
6.5
6.0
5.5
5.0
4.5
4.0
δ (ppm)

C_{10}

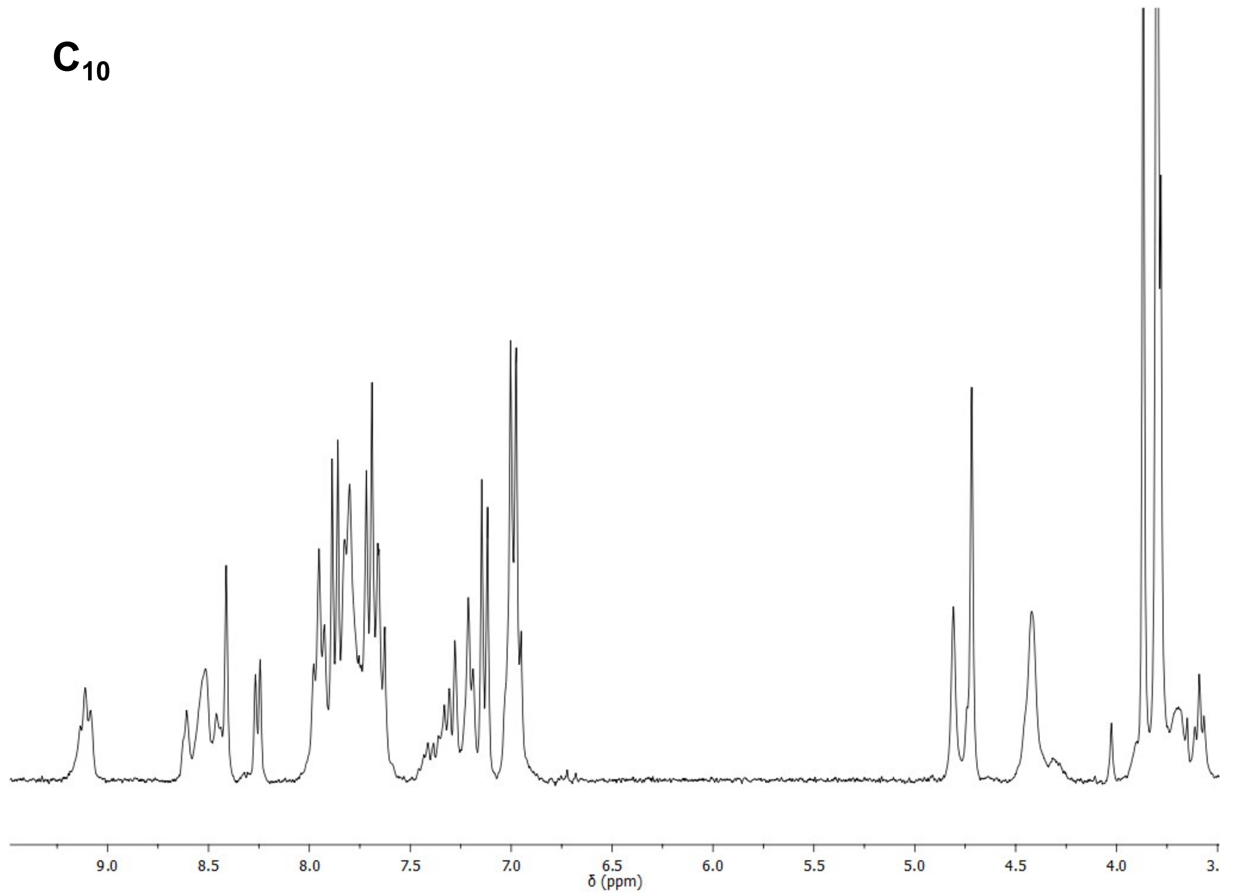
9.0
8.5
8.0
7.5
7.0
6.5
6.0
5.5
5.0
4.5
4.0
δ (ppm)

C_{11}

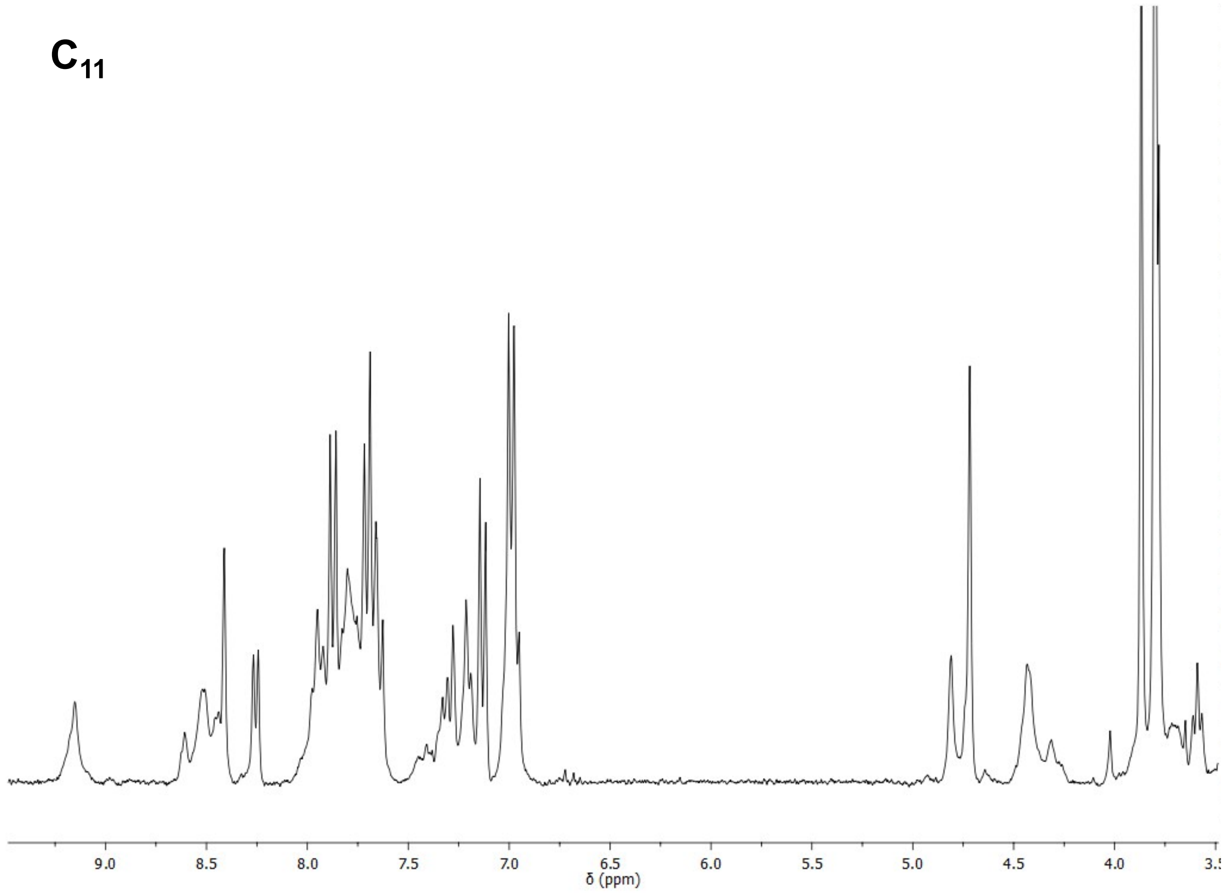
9.0
8.5
8.0
7.5
7.0
6.5
6.0
5.5
5.0
4.5
4.0
δ (ppm)

C_{12}

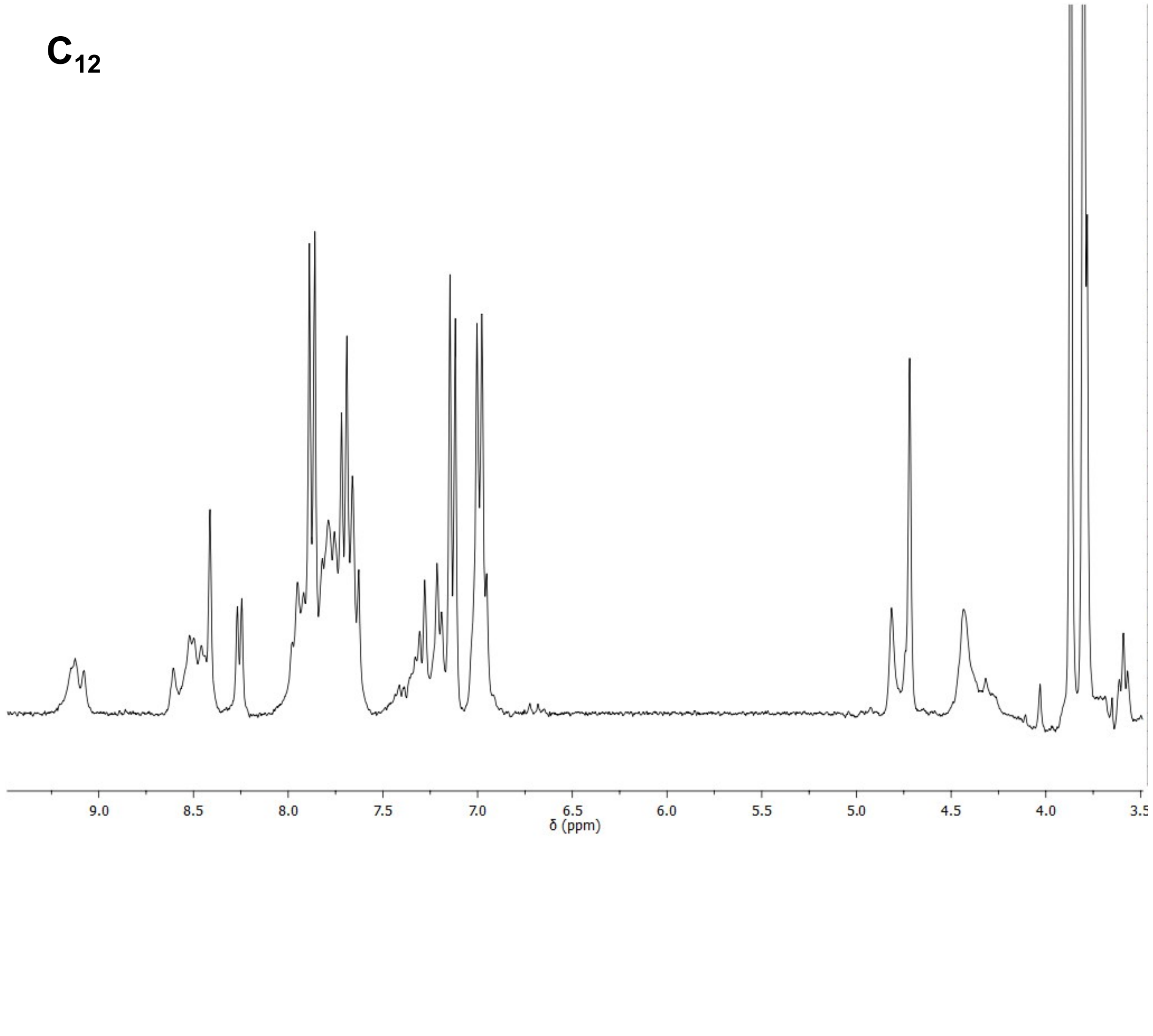
9.0
8.5
8.0
7.5
7.0
6.5
6.0
5.5
5.0
4.5
4.0
δ (ppm)

C_{13}

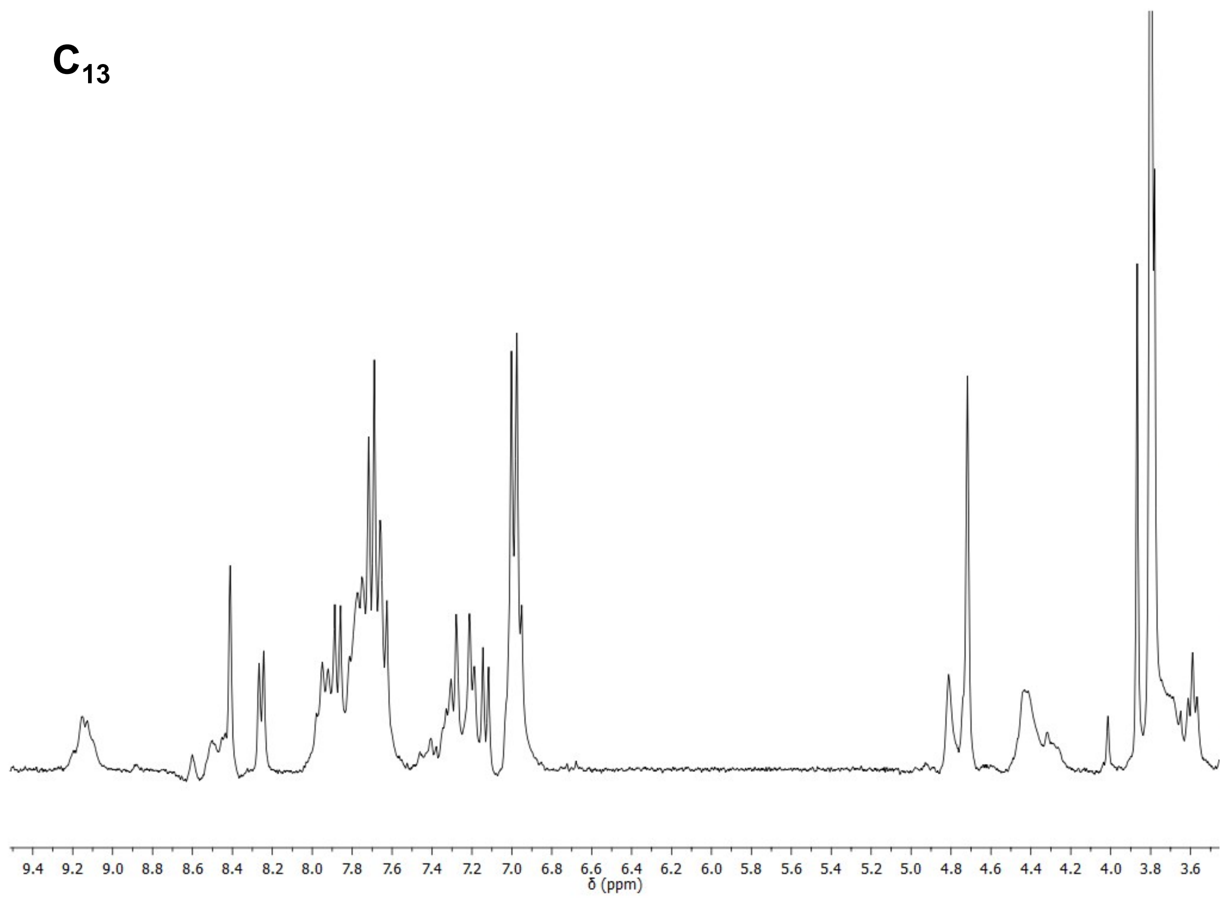

C_{14}

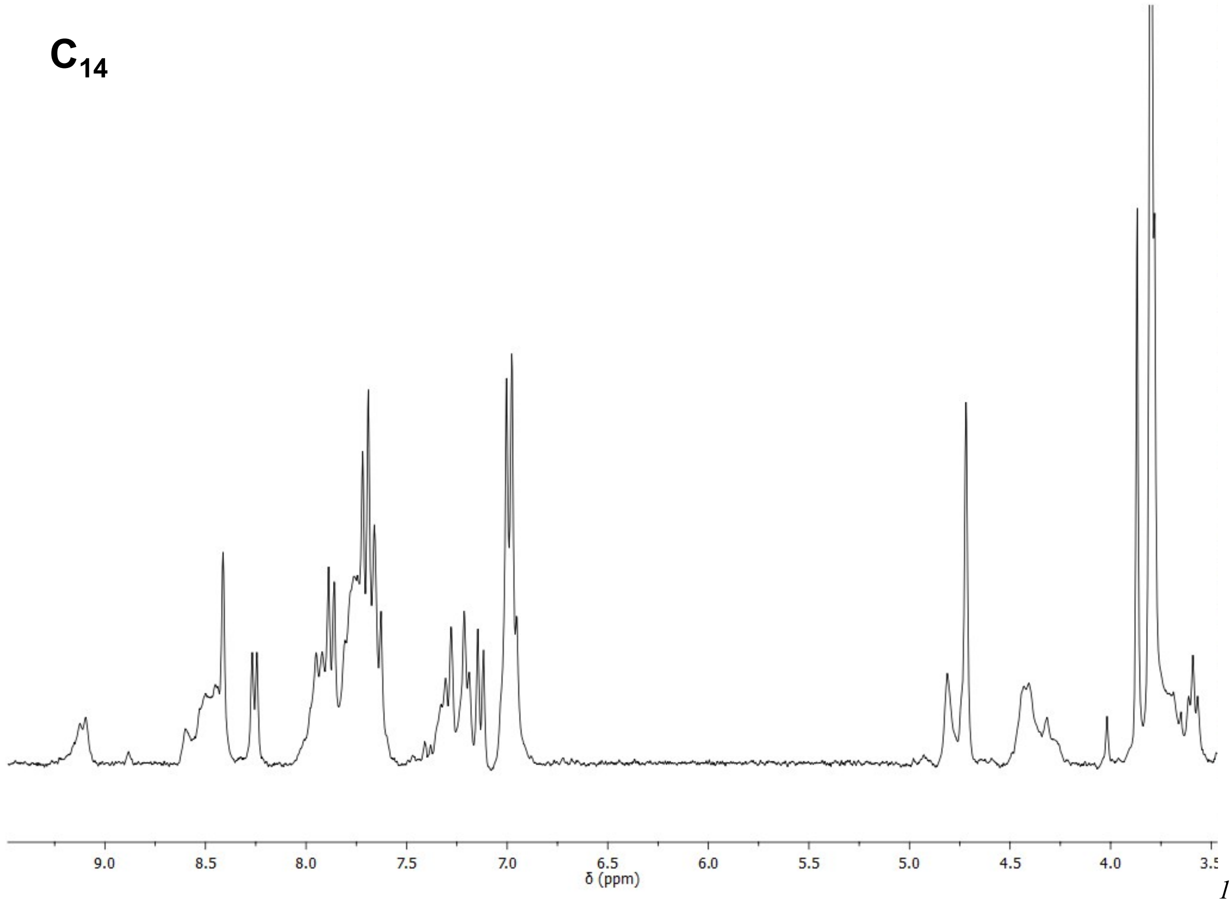

1

Figure S3 ^{1}H-NMR spectra of the **E+P+X** linkers experiments towards the guests series **C_5-C_{14}**

3.2 General procedure for Data analysis with PCA

The ESI-MS spectra were exported using the Data Analysis software of the ESI-MS and transformed into an excel sheet using OpenChrom. The peak intensities analysis was performed considering only the data corresponding to the *m/z* maximum of every di-charged peak clusters related to each cage, while the raw data analysis was performed choosing the spectral window containing all the di-charged peaks related to the **C_n@1(R-R'-R'')** species.
The ^{1}H NMR spectra were exported as ASCII using MestReNova taking into account the regions that presented the highest variance in the 3,8-9,5 ppm spectral window, which is diagnostic of the presence of the cages.
The experimental data collected by the two techniques were normalized internally, so that the highest point of intensity value for each experiment is considered at intensity equal to 1. In the case of ESI-MS data the mass of the guests **C_n** was previously removed. The data set was represented by a $n * m$ matrix X, where the n rows represent the experiments and the m columns represented the number of points acquired during the measurement.
The resulting set of data were run through the Principal Component Analysis (**PCA**) function of MatLabR2017a, using the following script:

$$[coeff, score, latent, \sim, explained] = pca(X)$$

The resulting PCA bidimensional plots were obtained from the score and coeff matrix from the pca function, considering the first and second principal components which account for at least 85% of the total variance of the data set.

3.2.1 PCA of ESI-MS spectra

The scatter plots of the two first principal components of the PCA of the data-set related to the DCL formed by **E-P-X** linkers. The peak intensity matrix is the matrix obtained from the normalized intensities of the ten peaks detected at ESI-MS. The corresponding Scores and Loadings plot of the first two components are showed in Figure S4 and Figure S6 respectively. The raw data matrix is generated from the normalized spectral data recorded by ESI-MS. The resulting scores plot is depicted in Figure S5.

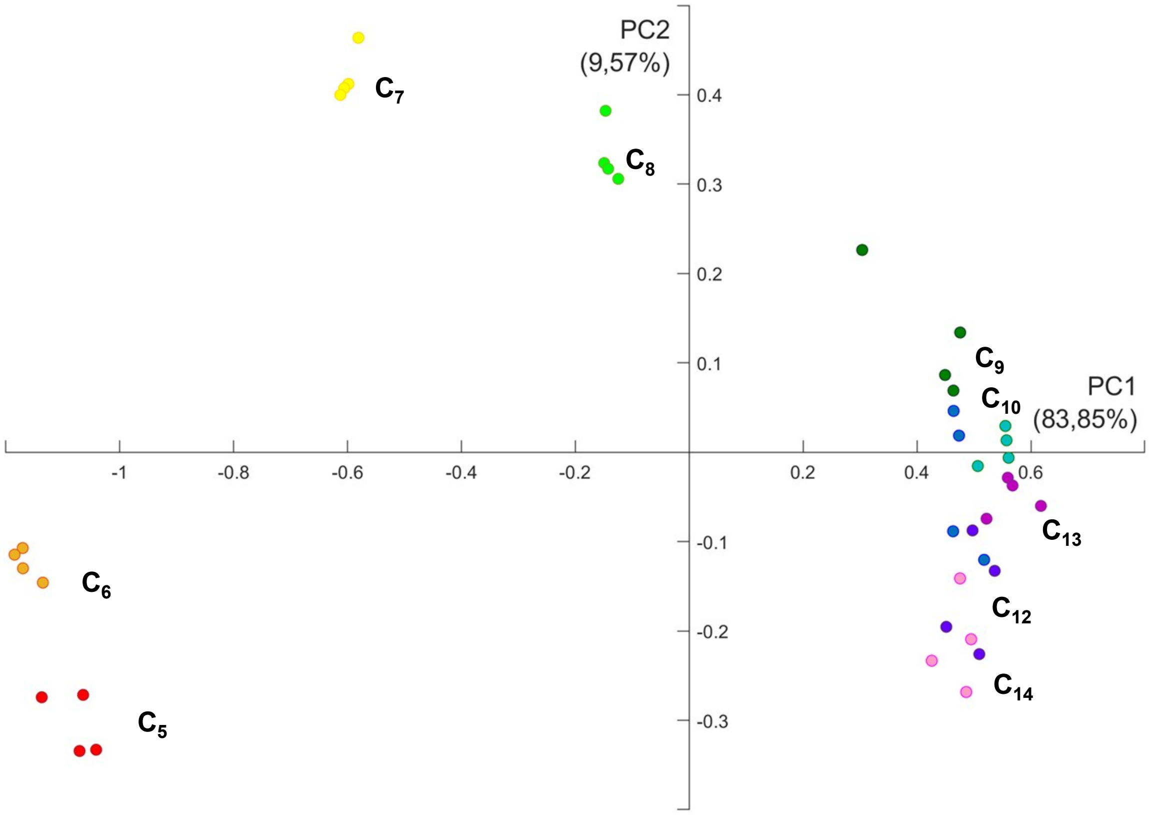

Figure S4 Scores plot of the first two principal components of the peak intensities matrix PCA

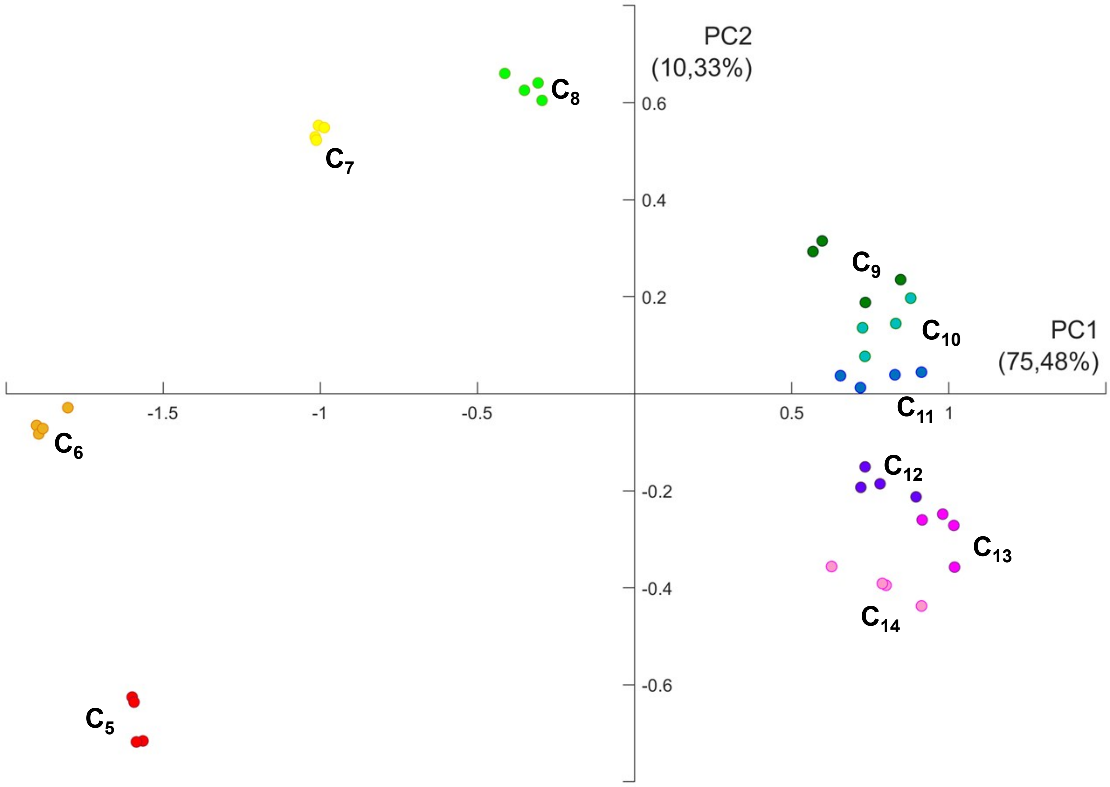

Figure S5 Scores plot of the first two principal components of the raw data matrix PCA

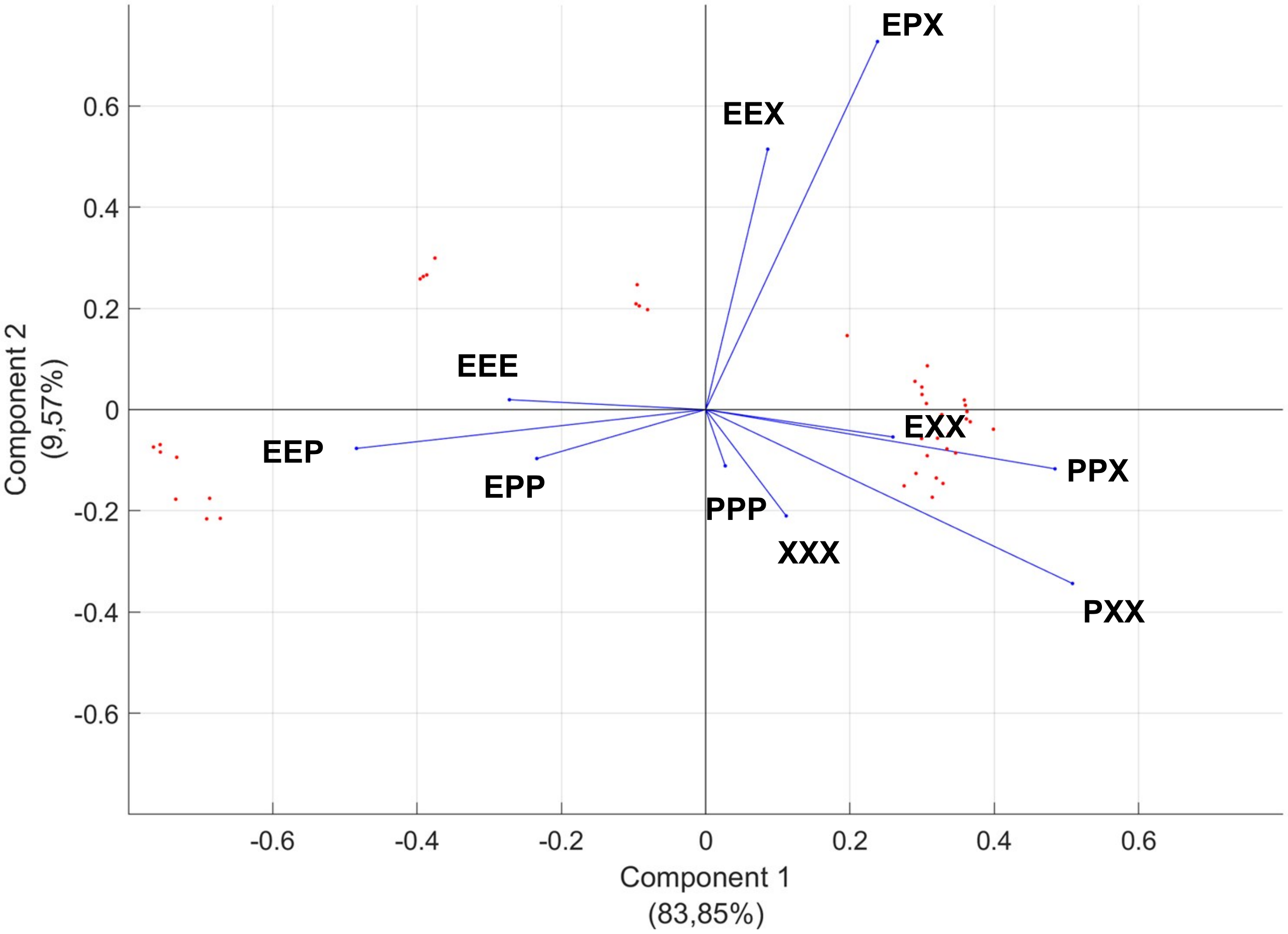

Figure S6 Loadings plot of the first two components of the peak intensities matrix PCA

3.2.2 PCA of ^{1}H-NMR spectra

The scatter plots of the two first principal components of the PCA of the data-set related to the experiment of **E+P+X** linkers. The raw data matrix is generated from the normalized spectral data. Each PCA considers a spectral window: 3,8-9,5 ppm (Figure S7); 7.7-7.9 ppm (Figure S8); 7.9-8.0 ppm (Figure S9); 8.3-8.6 ppm (Figure S10) and 8.9-9.3 ppm (Figure S11)

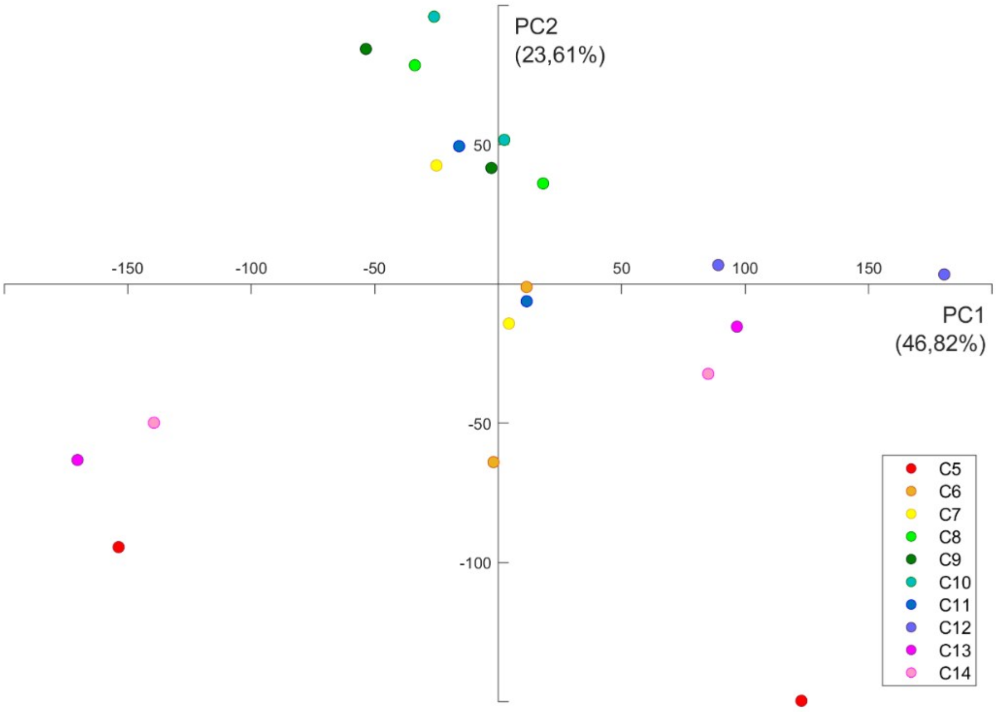

Figure S7 Scores plot of the first two principal components of the raw data matrix PCA for the spectral window 3.8-9.5 PCA

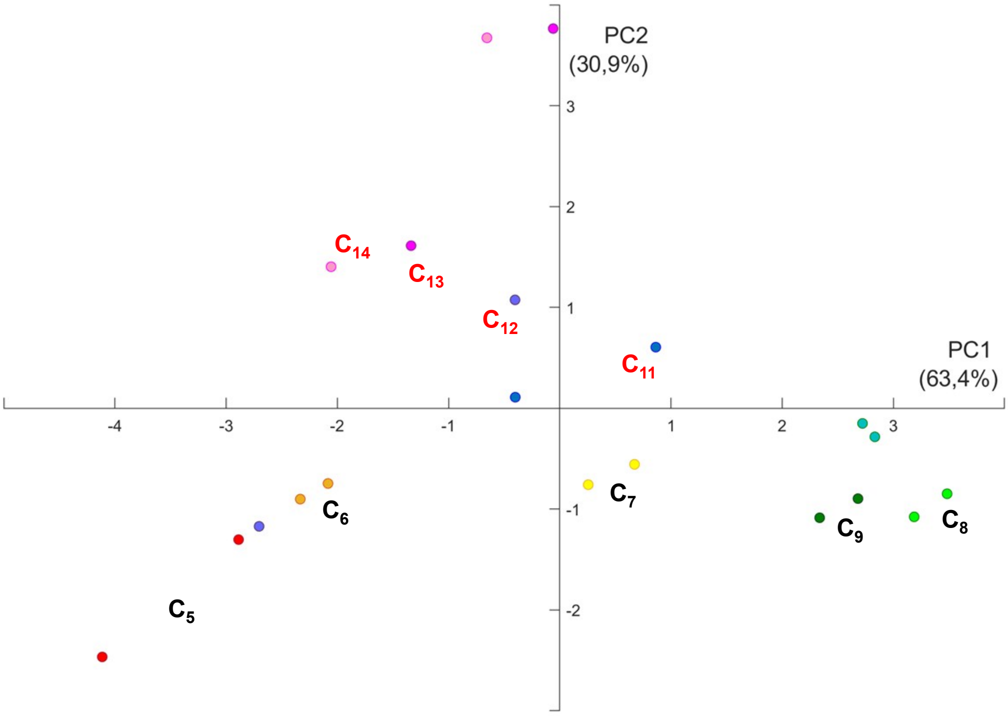

Figure S8 Scores plot of the first two principal components of the raw data matrix PCA for the spectral window 7.7-7.9 ppm. The points that are not correctly ordered are labelled in red.

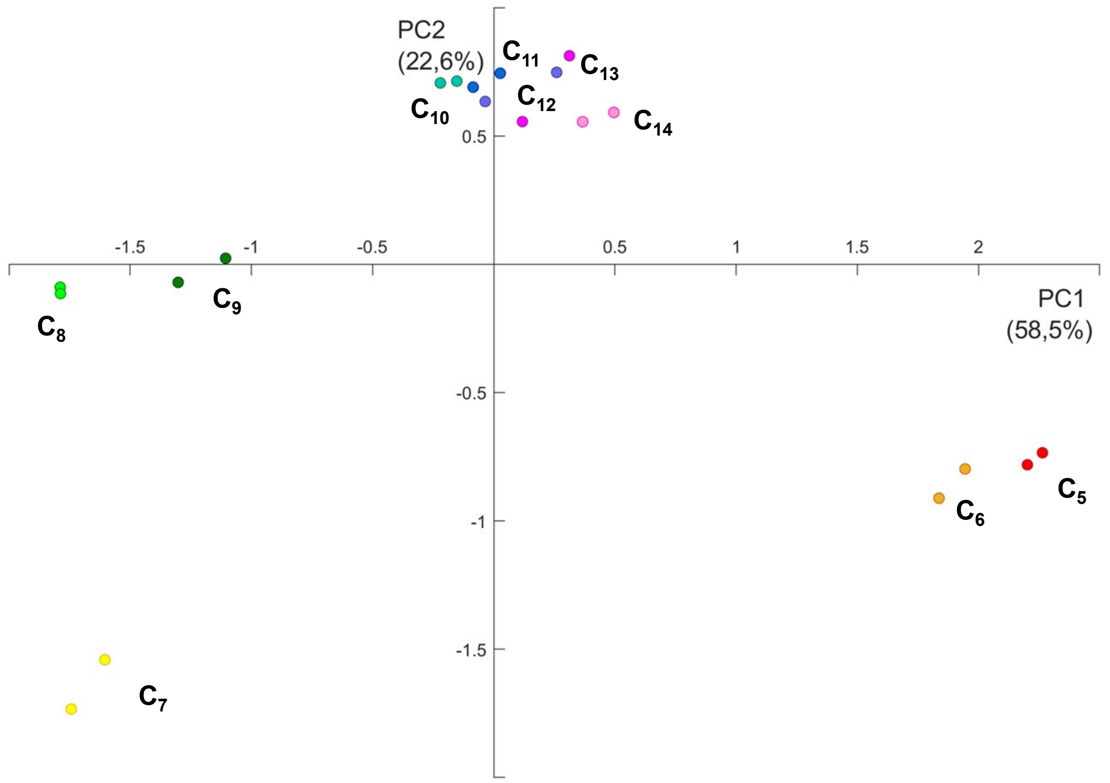

Figure S9 Scores plot of the first two principal components of the raw data matrix PCA for the spectral window 7.9-8.0 ppm.

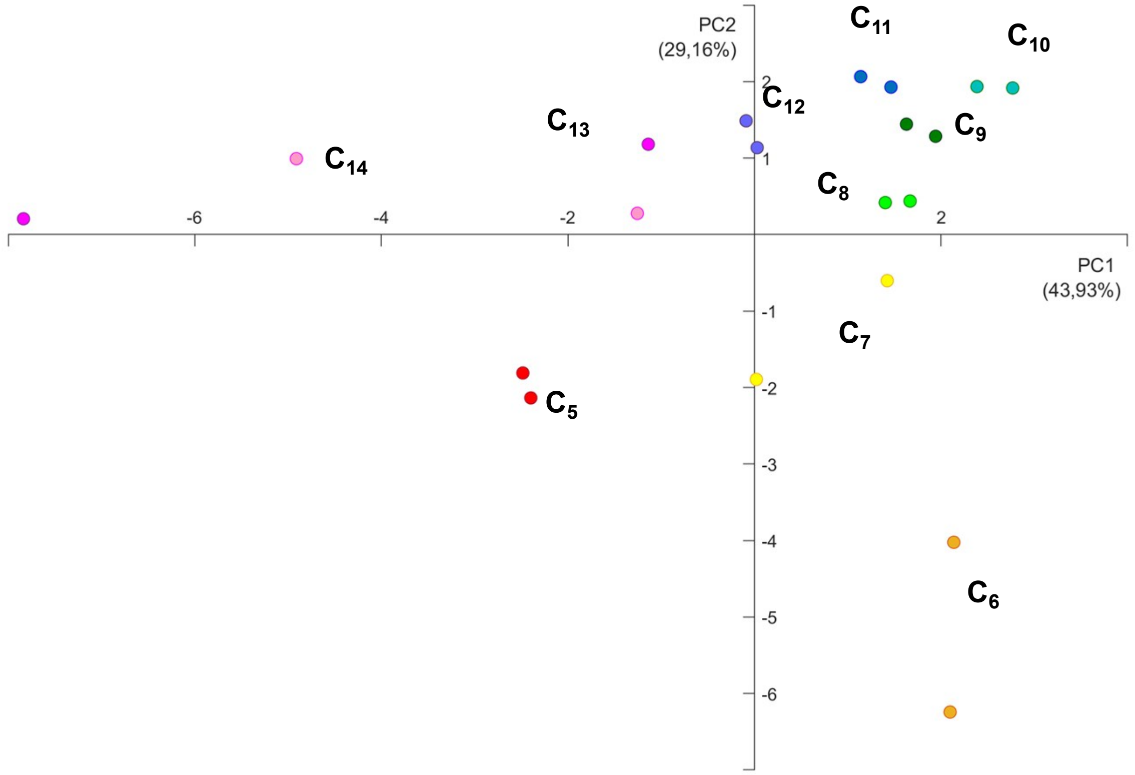

Figure S10 Scores plot of the first two principal components of the raw data matrix PCA for the spectral window 8.3-8.6 ppm.

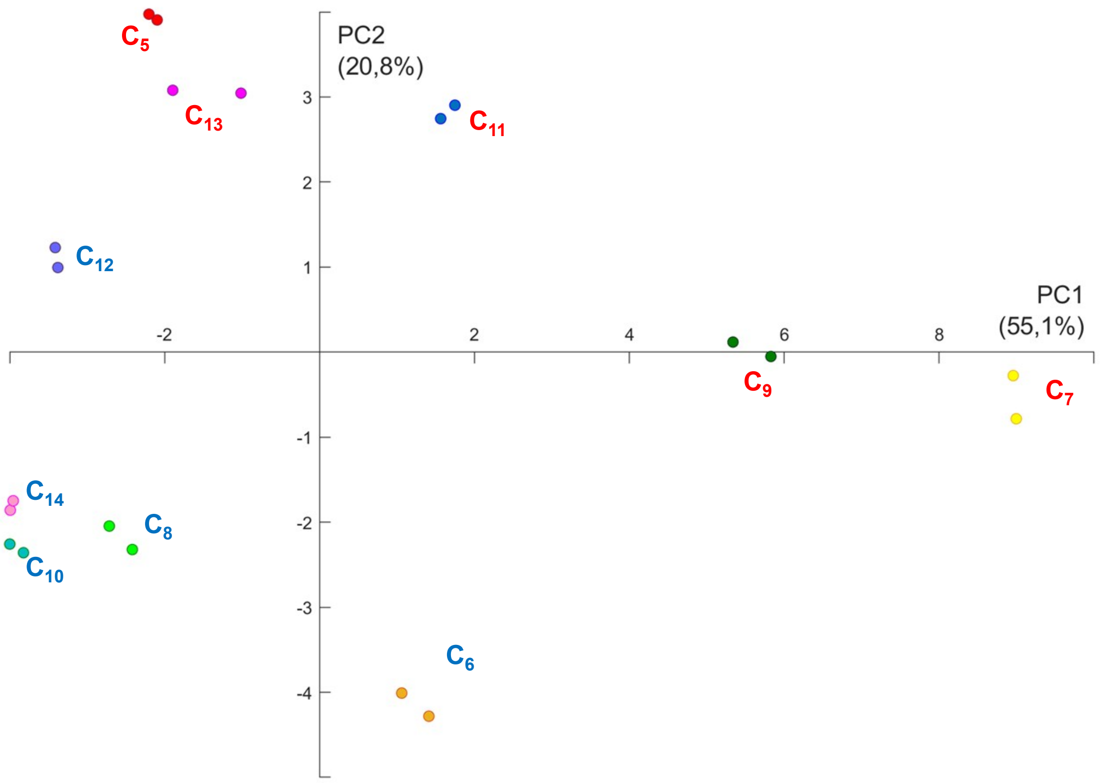

Figure S11 Scores plot of the first two principal components of the raw data matrix PCA for the spectral window 8.9-9.3 ppm. Points representing odd-length guests are labelled in red. Points representing even-length guests are labelled in blue.

3.3 Prediction of an unknown by projection in the PCA space

The new data were prepared as the previously described PCA data inputs. The original set of data were run through the Principal Component Analysis (**PCA**) function of MatLabR2018a. Afterwards, the unknown data were projected on the component space and compared to the original data using the following script:

$$[coeff,score] = pca(X);$$
$$coeff = diag(std(X))\backslash coeff;$$
$$[X, mu, sigma] = zscore(X);$$
$$sigma(sigma == 0) = 1;$$
$$x = bsxfun(@minus, x, mu);$$
$$x = bsxfun(@rdivide, x, sigma);$$
$$y = (x\backslash coeff');$$

With x being the new data and y the corresponding score value obtained by projection on the data covariance space. The resulting PCA bidimensional plots were obtained from the score and coeff matrix from the pca function, considering the first and second principal components which account for at least 85% of the total variance of the data set.

3.3.1 Projection of unknown in the PCA of ESI-MS spectra

The scatter plots of the two first principal components of the PCA of the data-set related to the DCL formed by **E-P-X** linkers. The peak intensity matrix is the matrix obtained from the normalized intensities of the ten peaks detected at ESI-MS. The corresponding Scores and Loadings plot of the first two components are showed in Figure S4 and Figure S6 respectively. The raw data matrix is generated from the normalized spectral data recorded by ESI-MS. The resulting scores plot is depicted in Figure S12.

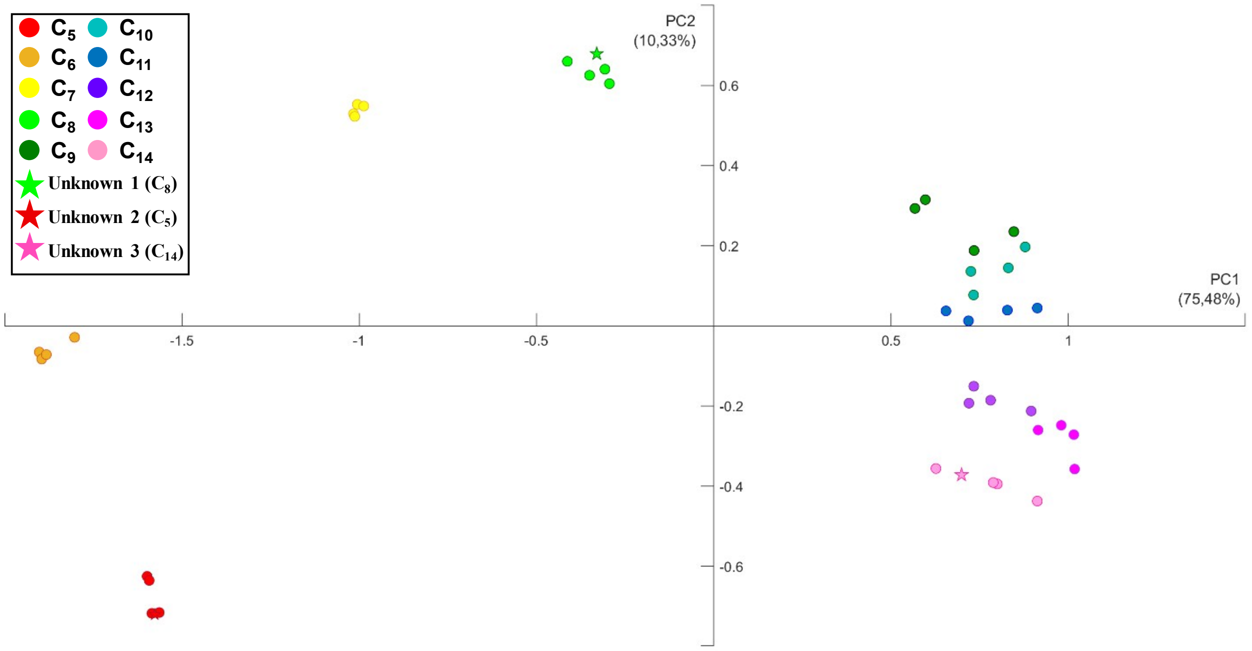

Figure S12 Scores plot of the first two principal components of the peak intensities matrix PCA with the projected additional data

3.3.2 Projection of unknown in the PCA of ¹H-NMR spectra

The scatter plots of the two first principal components of the PCA of the data-set related to the experiment of **E+P+X** linkers. The raw data matrix is generated from the normalized spectral data. Each PCA considers a spectral window: 7.9-8.0 ppm (Figure S13) and 8.9-9.3 ppm (Figure S14)

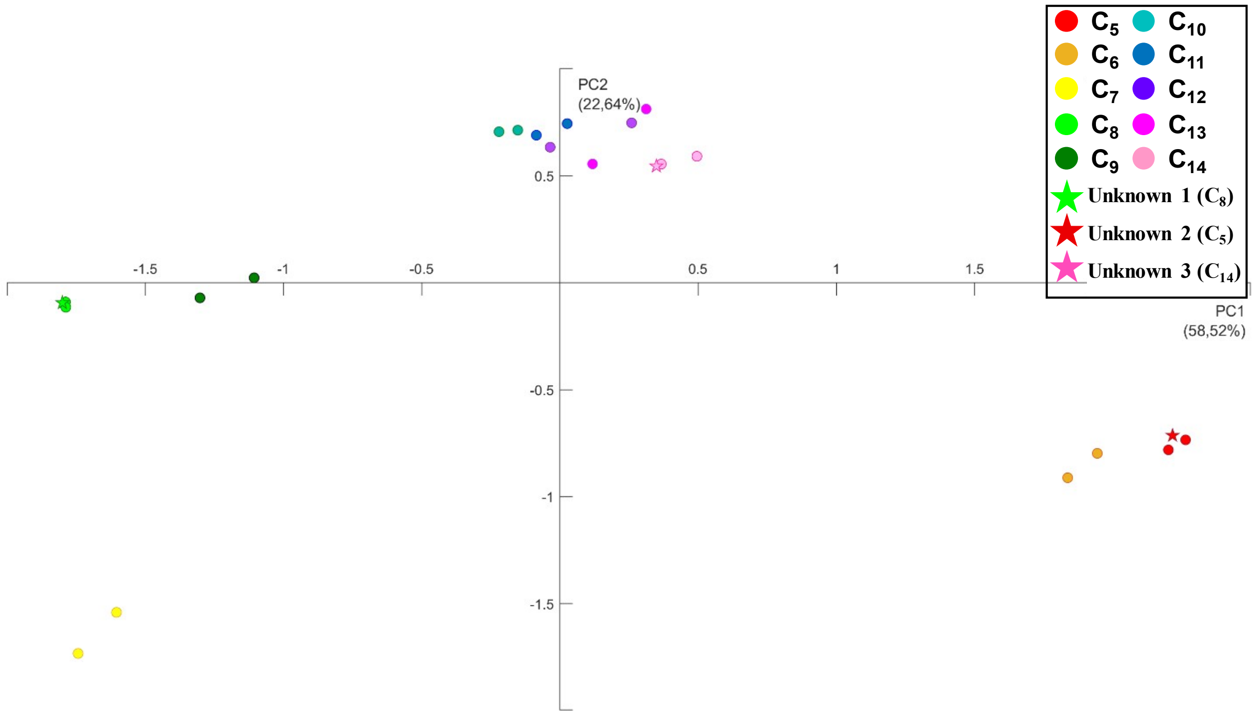

Figure S13 Scores plot of the first two principal components of the raw data matrix PCA for the spectral window 7.9-8.0, including the projections of the additional data points.

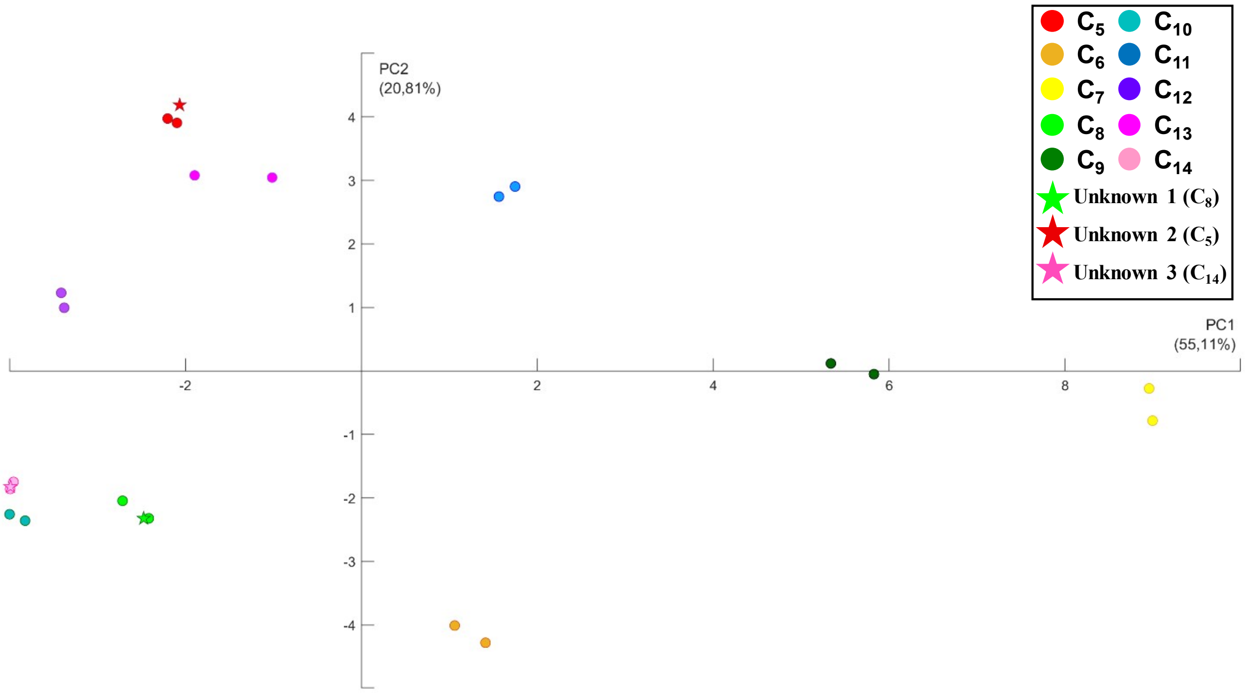

Figure S14 Scores plot of the first two principal components of the raw data matrix PCA for the spectral window 8.9-9.3 ppm, including the projection of the additional data points.
